# Nuclear matrix protein Matrin3 regulates alternative splicing and forms overlapping regulatory networks with PTB

**DOI:** 10.15252/embj.201489852

**Published:** 2015-01-19

**Authors:** Miguel B Coelho, Jan Attig, Nicolás Bellora, Julian König, Martina Hallegger, Melis Kayikci, Eduardo Eyras, Jernej Ule, Christopher WJ Smith

**Affiliations:** 1 Department of Biochemistry, University of CambridgeCambridge, UK; 2 Department of Molecular Neuroscience, UCL Institute of NeurologyLondon, UK; 3 MRC-Laboratory of Molecular BiologyCambridge, UK; 4 Computational Genomics, Universitat Pompeu FabraBarcelona, Spain; 5 Catalan Institute for Research and Advanced Studies (ICREA)Barcelona, Spain; 6 INIBIOMA, CONICET-UNComahueBariloche, Argentina

**Keywords:** alternative splicing, Matrin3, PTB

## Abstract

Matrin3 is an RNA- and DNA-binding nuclear matrix protein found to be associated with neural and muscular degenerative diseases. A number of possible functions of Matrin3 have been suggested, but no widespread role in RNA metabolism has yet been clearly demonstrated. We identified Matrin3 by its interaction with the second RRM domain of the splicing regulator PTB. Using a combination of RNAi knockdown, transcriptome profiling and iCLIP, we find that Matrin3 is a regulator of hundreds of alternative splicing events, principally acting as a splicing repressor with only a small proportion of targeted events being co-regulated by PTB. In contrast to other splicing regulators, Matrin3 binds to an extended region within repressed exons and flanking introns with no sharply defined peaks. The identification of this clear molecular function of Matrin3 should help to clarify the molecular pathology of ALS and other diseases caused by mutations of Matrin3.

## Introduction

Alternative splicing (AS) provides multi-cellular eukaryotes with a proteomic capacity that far exceeds the number of genes (Nilsen & Graveley, 2010). AS is an integral part of regulated programs of gene expression, often acting in concert with transcriptional control, but affecting different functionally related sets of genes (Blencowe, 2006). Regulation of AS is dictated primarily by RNA-binding proteins (RBPs) that can bind to specific RNA sequence elements and which can act as either activators of repressors (Coelho & Smith, 2014). Splicing predominantly occurs co-transcriptionally (Carrillo Oesterreich *et al*, 2011) in a chromatin context, and this temporal and spatial context provides additional layers of regulatory input into splicing decisions (Braunschweig *et al*, 2013). Nevertheless, RNA-binding proteins remain the key ‘readers’ of splicing codes (Barash *et al*, 2010). RBPs typically have one or more RNA-binding domains, and exhibit varying degrees of specificity, usually recognizing sequence motifs of ∽3–5 nt (Ray *et al*, 2013). While much has been learned about the action of individual RBPs binding to their cognate binding sites, the combinatorial nature of splicing regulation has led to an increased focus on the ways in which groups of regulatory proteins can act together (Barash *et al*, 2010; Campbell *et al*, 2012; Zhang *et al*, 2013; Cereda *et al*, 2014).

**Figure 1:**
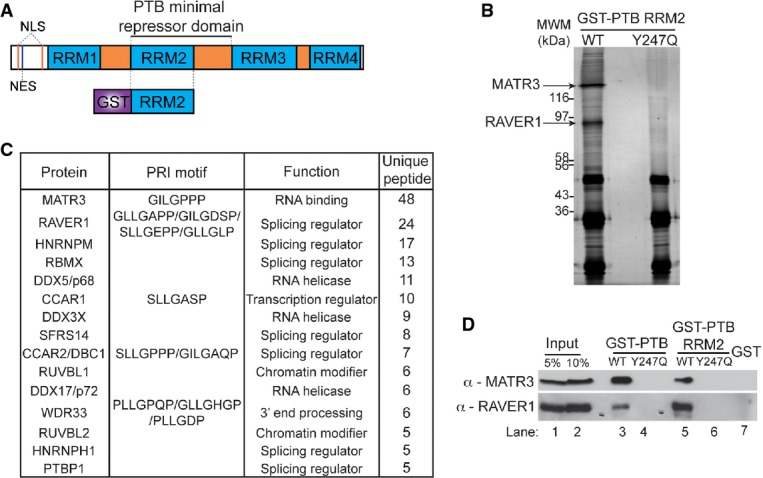
PTB RRM2 interacts with multiple RNA-binding proteins A Schematic representation of PTB (top) and the GST-PTB RRM2 (below), with the limits of the PTB minimal repressor domain indicated. PTB is composed of four RNA recognition motifs (RRM) with three linker regions in between them. It also contains a N-terminus bipartite nuclear localization signal (NLS) as well as a nuclear export signal (NES). The GST-PTB RRM2 is composed of the second RRM fused to a GST tag in the N-terminus.B Silverstain of the GST-PTB RRM2 pull-down of the wild-type RRM2 and the Y247Q mutant. Five microlitre of the pull-down was run on a 15% SDS–PAGE and silver-stained. Three strong bands can be seen which are due to the recombinant protein and the beads used for the pull-down. The region encompassing 50 kDa to the top of the gel was sliced and subjected to in-gel digestion and mass spectrometry. The two strongest bands visible in this region are labelled as Matrin3 and Raver1.C Proteins identified in the GST-PTB RRM2 pull-down ranked by their unique peptide number. The table shows the different proteins we found binding to RRM2, as well as when present, the sequence of the PRI motif. The indicated function is only a guideline as many have more functions than shown.D Western blot of the GST pull-down using antibodies against Matrin3 and Raver1. Lanes 1 and 2 show 5 and 10% of input, respectively, lanes 3 and 4 show GST-PTB full-length pull-down of wild-type and Y247Q mutant, respectively, and lanes 5 and 6 show GST-PTB RRM2 pull-down of wild-type and Y247Q mutant, respectively, and lane 7 with pull-down using GST alone. Source data are available online for this figure

Polypyrimidine tract binding (PTB/PTBP1/hnRNPI) protein is an intensively investigated RNA-binding protein, which regulates splicing and other post-transcriptional steps of gene expression (reviewed in Kafasla *et al*, 2012; Keppetipola *et al*, 2012; Sawicka *et al*, 2008). PTB binds to pyrimidine-rich motifs with core CU dinucleotides (Singh *et al*, 1995; Perez *et al*, 1997; Ray *et al*, 2013), and each of its four RRM (RNA recognition motif) family domains (Fig1A) can recognize such motifs (Oberstrass *et al*, 2005). Although primarily characterized as a repressive splicing regulator, it can also activate some splice sites and this has been related to differential positions of binding relative to regulated exons (Xue *et al*, 2009; Llorian *et al*, 2010). Although PTB can act alone as a regulator (Amir-Ahmady *et al*, 2005), genome-wide analyses suggest that it cooperates with a number of other proteins as a component of ‘tissue spicing codes’ (Castle *et al*, 2008; Wang *et al*, 2008; Barash *et al*, 2010; Bland *et al*, 2010; Llorian *et al*, 2010). Structure-function analysis has indicated that despite their similar RNA-binding preferences, the four RRMs of PTB show functional diversification (Liu *et al*, 2002; Robinson & Smith, 2006; Mickleburgh *et al*, 2014). Of particular importance for synergistic action with other regulators, RRM2 can interact with both RNA via its canonical β-sheet surface, and with short linear PRI (PTB-RRM Interaction) motifs found in the co-regulator Raver1 (Rideau *et al*, 2006; Joshi *et al*, 2011). The PRI motif is defined by the consensus sequence [S/G][IL]LGxΦP and binds to the dorsal surface of PTB RRM2, with Tyr247 of PTB particularly critical for this interaction (Rideau *et al*, 2006; Joshi *et al*, 2011). PTB RRM2, along with the following linker sequence, is sufficient for splicing repressor activity when artificially tethered as an MS2 fusion protein (Robinson & Smith, 2006) (Fig1A). Despite the fact that Raver1 can act with PTB as a co-regulator of *Tpm1* splicing (Gromak *et al*, 2003; Rideau *et al*, 2006), Raver1 null mice showed no alteration in *Tpm1* splicing (Lahmann *et al*, 2008) and knockdown of Raver1 in HeLa cells showed only a few changes in alternative splicing (Hallegger *et al*, manuscript in preparation). Therefore, it remains possible that other co-regulatory proteins with PRI motifs might interact with PTB RRM2.

**Figure 2:**
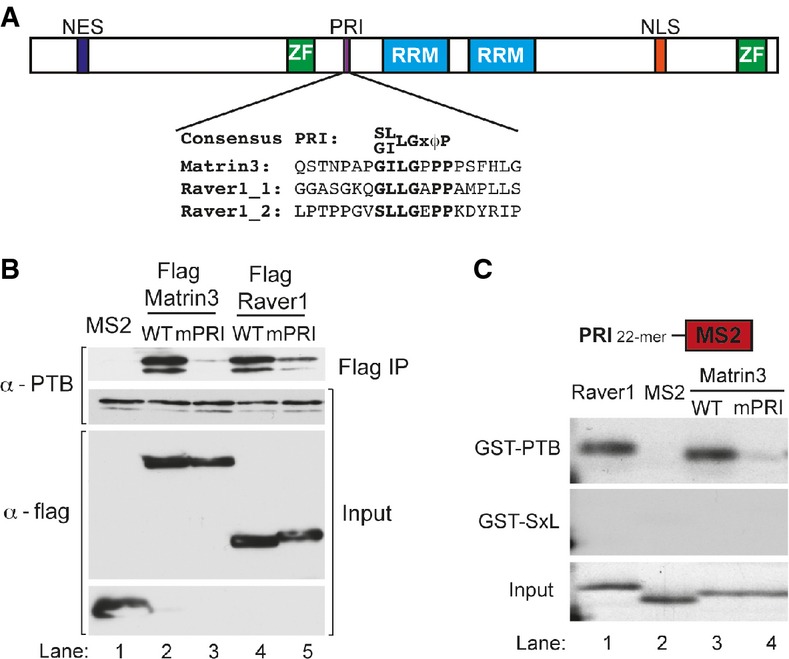
Matrin3 interacts with PTB via a PRI motif A Schematic representation of Matrin3. Matrin3 is composed by two zinc finger (ZF) domains, two tandem RNA recognition motifs (RRM), as well as a N-terminal nuclear export signal (NES) and a C-terminal nuclear localization signal (NLS). A PRI motif is localized between the first ZF and the first RRM, and the sequence is aligned with the sequence from the two functional PRIs from Raver1. Conserved PRI residues are in bold.B FLAG immunoprecipitation of Matrin3 and Raver1, both with wild-type and with PRI mutated, and FLAG-MS2 as a negative control. The immunoprecipitated complex was separated in a SDS–PAGE and subjected to Western blot using antibody against PTB which showed interaction to wild-type Matrin3 (lane 2) and Raver1 (lane 4). The input was also analysed by Western blot with antibodies against PTB as a loading control and against the FLAG tag to ensure equal expression of proteins.C The 20-residue Raver1 491–511 (lane 1) and the Matrin3 346–365 (lane 3) peptide fused to MS2 were transcribed and translated *in vitro* (Input) and then pulled down with GST-PTB or with GST-SXL as a control. Effects of single mutation of the Matrin3 PRI GILPPP to GIAPPP were also tested (lane 4). Source data are available online for this figure

Matrin3 is one of the most abundant inner nuclear matrix proteins (Nakayasu & Berezney, 1991). The main isoforms of Matrin3 are over 800 amino acids in size, but most of the protein is not comprised of structurally characterized domains, with the exception of two DNA-binding C2H2 zinc finger (ZF) and two RRM domains (Hibino *et al*, 2006), and a bi-partite nuclear localization signal (NLS) (Hisada-Ishii *et al*, 2007) (Fig2A). Matrin3 is essential for viability of some cells (Hisada-Ishii *et al*, 2007; Przygodzka *et al*, 2010), and alterations in Matrin3 levels are associated with some diseases (Bernert *et al*, 2002; Bimpaki *et al*, 2009). Moreover, missense mutations in Matrin3 have been associated with asymmetric myopathy with vocal cord paralysis (Senderek *et al*, 2009) and amyotrophic lateral sclerosis (ALS) (Johnson *et al*, 2014). Matrin3 is located diffusely throughout the nucleoplasm and is concentrated in the nuclear scaffold (Zeitz *et al*, 2009), and its DNA- and RNA-binding domains suggest that it may play roles in processes associated with the nuclear matrix or nucleoplasm. It can anchor chromosomes to the nucleus matrix by binding to the MAR/SAR elements (Hibino *et al*, 1992). Introduction of MAR/SAR sites upstream of a promoter stimulates transcription, suggesting Matrin3 binding to these elements might promote transcription (Hibino *et al*, 2000), a suggestion supported by the proximity of Matrin3 with RNA Pol II promoters (Malyavantham *et al*, 2008) and enhancers (Skowronska-Krawczyk *et al*, 2014). Matrin has also been shown to be involved in the early stages of the DNA double-strand break response (Salton *et al*, 2010). A number of functional roles associated with cellular and viral RNA have been suggested for Matrin3 including mRNA stabilization (Salton *et al*, 2011), nuclear retention of hyperedited RNA (Zhang & Carmichael, 2001) and Rev-dependent export of unspliced HIV1 RNA in conjunction with PTB-associated factor (PSF) (Kula *et al*; Kula *et al*, 2011; Yedavalli & Jeang, 2011). Matrin3 interacts with a number of splicing regulators including hnRNPK (Salton *et al*, 2011), hnRNPL, SFRS7, p68 (Zeitz *et al*, 2009), NOVA-1/-2 (Polydorides *et al*, 2000), CTCF (Fujita & Fujii, 2011; Shukla *et al*, 2011), as well as the transcription machinery itself (Das *et al*, 2007). Despite the interactions with splicing factors, there is no direct evidence for Matrin3 functioning as a splicing regulator.

Here, we set out to identify nuclear proteins that interact with PTB RRM2. Matrin3 was the major interacting protein in HeLa nuclear extracts, interacting via a single PRI motif that is necessary and sufficient for interaction. Using RNAi knockdown and splice-sensitive microarray analysis in conjunction with iCLIP of Matrin3 and PTB, we find that Matrin3 acts widely as a splicing regulator. While a number of its target splicing events are shared with PTB, the majority are PTB independent and involve Matrin3 action as a repressor. Matrin3 binding was observed in the introns flanking repressed exons, but in contrast with other splicing regulators, the binding occurred to an extended region with no clear peaks. Structure-function analysis indicates that Matrin3 splicing activity requires both the RRM domains and the PRI, even for ASEs that are not co-regulated by PTB.

## Results

### Identification of PTB RRM2 binding partners

With the aim of understanding better the function of the minimal PTB repressor domain, we carried out a proteomic screen to identify interacting protein partners of PTB RRM2, the main component of the repressor domain (Robinson & Smith, 2006). PTB RRM2 was fused to GST in wild-type (WT) and Y247Q mutant form, which impairs interaction with Raver1 PRI peptides (Joshi *et al*, 2011) (Fig1A), and used as bait to pull down interacting proteins from HeLa nuclear extracts. Numerous proteins bound to WT RRM2 but not the Y247Q mutant (Fig1B). Proteins pulled down by WT GST-RRM2 were identified by mass spectrometry (Fig1C and Supplementary Table S1). They include RNA-binding proteins (MATR3, RAVER1, HNRNPM, RBMX, DDX5, DDX3X, SFRS15, DDX17, HNRNPH1 and PTB itself), proteins with role in transcription regulation (CCAR1, KIAA1967/CCAR2 and RUVBL1/2) and a protein found in 3′ end processing complexes (WDR33). Four of the five unique PTB peptides are located within RRM2 and so could derive from the bait protein. Proteins with roles in transcription and 3′ end processing may present links to unknown activities of PTB in the case of transcription regulation, and in the case of WDR33 (Shi *et al*, 2009), a molecular link to an already reported function of PTB in 3′ end processing (Moreira *et al*, 1998; Castelo-Branco *et al*, 2004).

The strongest protein interaction detected, as indicated by number of unique peptides and MASCOT score, was the nuclear matrix protein Matrin3, followed by Raver1 (Fig1C and D). These two proteins correspond to the major protein bands interacting specifically with WT but not mutant RRM2 (Fig1B, arrows). Matrin3, CCAR1, KIAA1967 and WDR33 all have potential PRI motifs similar to those in Raver1 (Figs1C and 2A). We validated the Matrin3-PTB interaction by Western blot of GST-RRM2 and full-length GST-PTB pull-downs, comparing wild-type (WT) and Y247Q mutant proteins (Fig1D). Both Matrin3 and Raver1 interacted strongly with GST-RRM2 and GST-PTB proteins, and in both cases, the Y247Q mutation abolished the interaction. This indicates that the RRM2 interaction is sufficient and also necessary in the context of full-length GST-PTB for interaction with Matrin3 and Raver1 (Fig1D). However, while Matrin3 interacted equally well with RRM2 or full-length PTB, Raver1 interacted more strongly with full-length PTB, suggesting that other regions of PTB may also contact Raver1.

### Matrin3 PRI motif is necessary and sufficient for PTB interaction

Matrin3 is a large nuclear protein with 847 amino acids that can bind both to DNA via two C2H2 zinc finger domains (ZF1 and ZF2) and to RNA by its tandem RNA recognition motifs (RRM1 and RRM2) (Hibino *et al*, 2006). A potential PRI motif, GILGPPP, is located between ZF1 and RRM1. This matches the PRI consensus (Fig2A) and is located in a disordered region, which is important for the function of short linear motifs (Dinkel *et al*, 2014). Moreover, the motif is absolutely conserved across 84 mammalian, avian, reptilian and amphibian species (UCSC browser, Vertebrate Multiz Alignment & Conservation, 100 Species). In order to test whether the GILGPPP motif is functional, we mutated it to GAAAPPA (mutated residues underlined) in a FLAG-tagged Matrin3 expression vector and tested the effect on PTB binding by anti-FLAG co-immunoprecipitation. As control, we used wild-type Raver1 and a mutant with all four PRI motifs mutated (Rideau *et al*, 2006). FLAG-tagged Matrin3 and Raver1 both co-immunoprecipitated PTB (Fig2B). Mutation of the single PRI in Matrin3 nearly eliminated PTB co-immunoprecipitation (lane 3), a more emphatic effect than mutation of the Raver1 PRI motifs (Fig2B, lane 5). We next tested whether the Matrin3 PRI is sufficient for binding to PTB. We *in vitro* transcribed and translated the Matrin3 and the Raver1_1 PRIs (Fig2A) fused to the bacteriophage MS2 coat protein. Both the Raver1 and Matrin3 peptides were pulled down by GST-PTB (Fig2C, lanes 1, 3). As negative controls, no binding was observed to an unrelated RNA-binding protein, GST-SXL, and MS2 alone was not pulled down by GST-PTB or GST-SXL (lane 2). The specificity of the interaction was demonstrated by mutation to alanine of the conserved leucine-3 of the PRI, which strongly impaired binding to PTB (Fig2C, lane 4). These data therefore demonstrate that the PRI motif of Matrin3 is both necessary and sufficient for interaction with PTB.

### Matrin3 is a widespread regulator of alternative splicing

**Figure 3:**
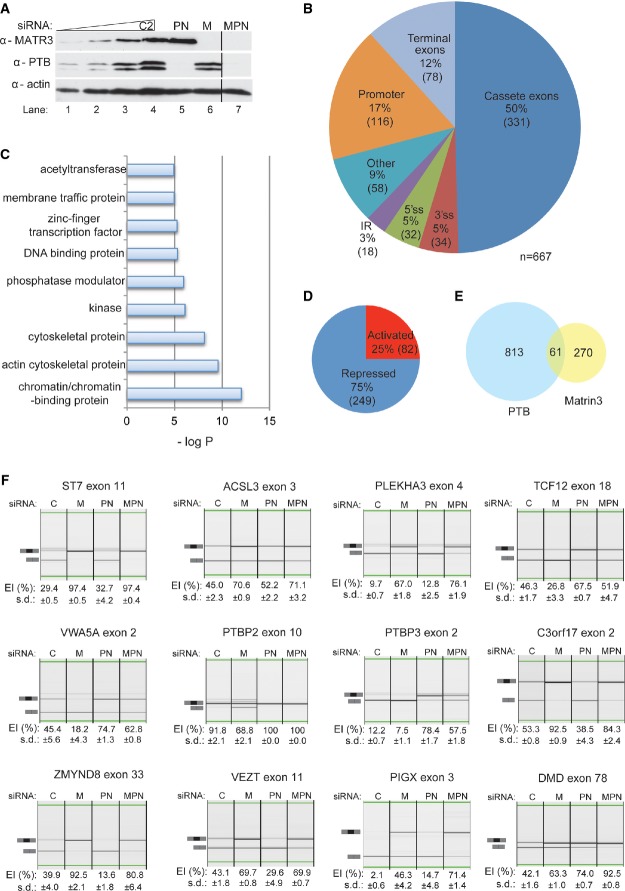
Global splicing effects upon Matrin3 knockdown A Western blot probed for Matrin3 (top panel), PTB (middle panel) and actin (lower panel). Lanes 1–4 contain a twofold dilution of the control C2 sample (lane 1—12.5%, lane 2—25%, lane 3—50%, lane 4—100%). Lanes 4–7 contain equal amount of proteins, as can be confirmed by the anti-actin (lower panel), of control sample (lane 4), double-PTB and nPTB siRNA-treated sample (lane 5), Matrin3 siRNA-treated sample (lane 6) and triple knockdown of Matrin3, PTB and nPTB (lane 7). Lane 7 is from the same gel and exposure, but some lanes present in the original gel were cropped for clarity, and a black line to indicate cropping was placed.B Pie chart of the different categories of Matrin3-regulated alternative splicing events (ASEs), 330 cassette exons (50%), 116 alternative promoter usage (17%), 78 terminal exons (11%), 32 alternative 5′ (5%) and 34 3′ (5%) splice site and 18 intron retention (IR; 3%) events.C Gene ontology (GO) analysis of the Matrin3-regulated cassette exons. The *x*-axis represents the *P*-value in a logarithmic scale as shown.D Pie chart of the activated and repressed cassette exons by Matrin3.E Venn diagram of the overlap between the PTB- (blue) and Matrin3- (yellow) regulated cassette exons, showing the 813 events regulated only by PTB, 270 by Matrin3 only and the 61 that overlap.F RT–PCR validation of Matrin3-regulated alternative spicing events in the ST7, ACSL3, PLEKHA3, TCF12, VWA5A, PTBP2, PTBP3, C3orf17, ZMYND8, VEZT, PIGX and DMD genes. In each case, triplicates for each condition (C—control, M—Matrin3, PTB/nPTB and Matrin3/PTB/nPTB siRNA transfection samples) were analysed and exon inclusion (EI) percentage is shown beneath the corresponding lane, along with the standard deviation (s.d.). Source data are available online for this figure

The interaction of Matrin3 with PTB led us to hypothesize that it may play a role in the co-regulation of some PTB-regulated alternative splicing events (ASEs). To test this hypothesis, we transfected HeLa cells with siRNAs targeting the Matrin3 mRNA and observed a > 90% decrease in the Matrin3 protein levels (Fig3A). Total RNA from knockdown and control samples was purified and analysed using Human Junction microarrays (HJAY), containing probe sets for all annotated human exons and exon–exon junctions (Llorian *et al*, 2010). The array data were analysed using the ASPIRE3 pipeline. Only 61 genes showed changes in RNA levels of greater than twofold, including the expected reduction of Matrin3 levels (3.7-fold; Supplementary Table S2). This suggests, in contrast to a previous report (Salton *et al*, 2011), that Matrin3 does not play a widespread role in stabilizing mRNAs. We did observe down-regulation of some of the previously reported mRNAs (Salton *et al*, 2011), but also observed alteration of alternative splicing events towards isoforms of these mRNAs with premature termination codons, which normally leads to nonsense-mediated decay (see Discussion).

Next, we examined the potential role of Matrin3 in regulating alternative splicing. Significant changes in splicing were predicted by ASPIRE using a threshold of |dIrank| > 1 (Supplementary Table S3), which has previously been shown to produce a validation rate of > 80% (König *et al*, 2010; Wang *et al*, 2010). This identified 667 ASEs, half of which were cassette exons (*n* = 331; 50%; Fig3B). Of the Matrin3-regulated cassette exons, 75% showed increased inclusion upon Matrin3 knockdown, indicating that Matrin3 represses inclusion of these exons (Fig3D). Notably, the degree of confidence in the changes observed in splicing of the 25% Matrin3-activated cassette exons was lower when compared to the Matrin3-repressed ones (Supplementary Fig S1).

We next examined the cassette exons that may be jointly regulated by Matrin3 and PTB, using the HJAY data set produced upon knockdown of PTB and PTBP2 (Llorian *et al*, 2010). The double knockdown is essential as upon PTB knockdown, its neuronal paralogue PTBP2 is upregulated and can partially compensate for loss of PTB (Boutz *et al*, 2007; Makeyev *et al*, 2007; Spellman *et al*, 2007). Only 61 (18.4%) of the 331 Matrin3-regulated cassette exons were also regulated by PTB (Fig3E). While the number of co-regulated cassette exons is 2.2-fold greater than expected by chance (expected 27.4, *P* = 5.5e^−10^, hypergeometric test), the majority of the Matrin3-regulated ASEs are PTB independent.

We validated a number of the cassette exon events predicted to be regulated by Matrin3 and PTB by knockdown of Matrin3 or PTBP1/PTBP2. We also tested the effects of combined knockdown of Matrin3/PTBP1/PTBP2 (Fig3A). RT–PCR was carried out using primers in flanking constitutive exons and the percentage exon inclusion determined (Fig3F). Four different classes of events were observed, depending on their response to Matrin3 and PTB knockdown: Matrin3 repressed, PTB independent (ST7 exon 11, ACSL3 exon 3 and PLEKHA3 exon 4); Matrin3 activated, PTB repressed (TCF12 exon 18, VWA5A exon 2, PTBP2 exon 10 and PTBP3 exon 2); Matrin3 repressed, PTB activated (C3orf17 exon 2, ZMYND8 exon 33 and VEZT exon 11); and repressed by both Matrin3 and PTB (PIGX exon 3 and DMD exon 78). In the cases where Matrin3 and PTB activities were opposed, knockdown of the repressor protein had a larger effect and tended to be dominant over the activator. In some cases, knockdown of the activator had no effect in the absence of the repressor (e.g. VEZT exon 11 and PTBP2 exon 10), suggesting that that the sole function of the activator is to antagonize the repressor.

### Properties of Matrin3-regulated exons

**Figure 4:**
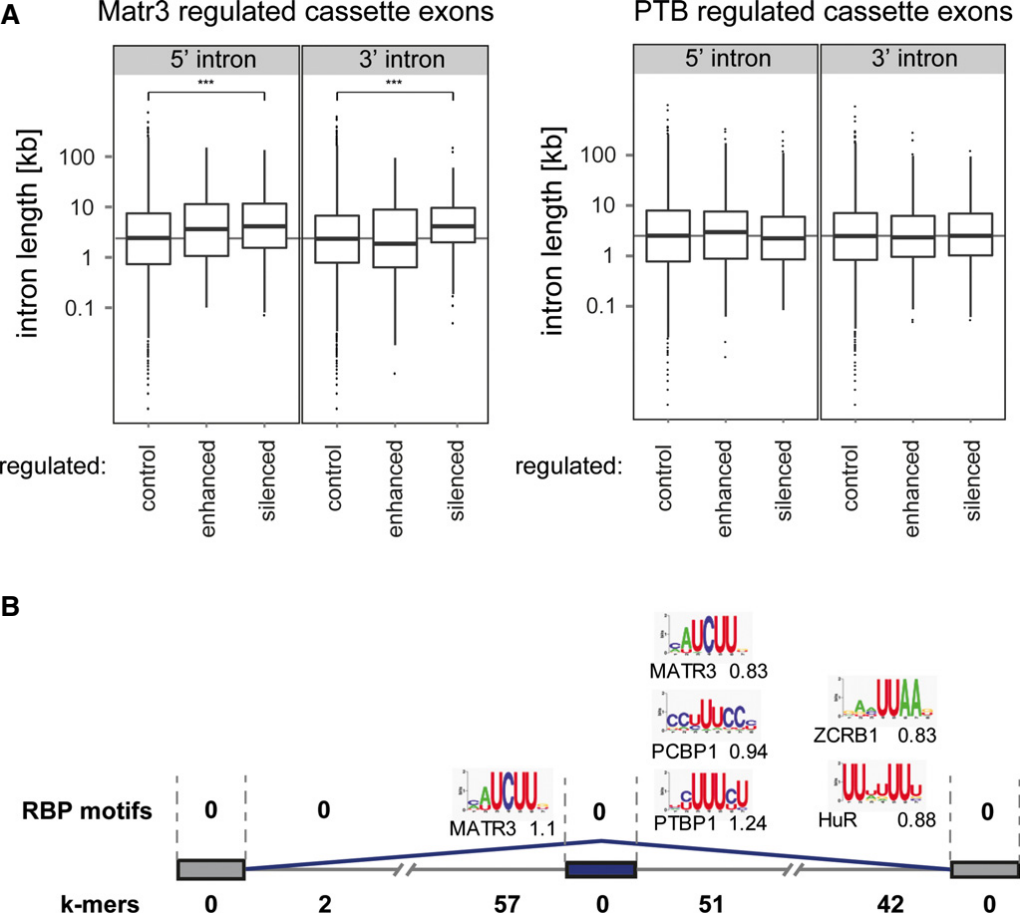
Bioinformatic analysis of Matrin3-regulated splicing events A Intron lengths flanking exons regulated by Matrin3, PTB or control exons. Kruskal–Wallis rank-sum test was used to test for significant changes (Matr3 *P*-value = 3.947e^−15^, PTB *P*-value = 0.3896). ***P < 0.001.B Diagram of Matrin3-repressed cassette exons with enriched RBP motifs for human proteins (Ray *et al*, 2013) shown above and the number of enriched pentamers shown below in each of 7 locations. The RBP motifs are shown with their consensus binding site logo (Ray *et al*, 2013) and the respective motif enrichment score (odds ratio). The significant enriched k-mers are shown in Supplementary Table S4, and all enriched RBP motifs (multiple species) are shown in Supplementary Table S5.

In order to assess whether the exons regulated by Matrin3 possess any specific splicing features, we examined 5′ and 3′ splice sites, branch points, pyrimidine tracts, and nucleotide composition (Supplementary Fig S3) and flanking intron lengths (Fig4A). Few significant differences were observed compared to a control set of annotated cassette exons unaffected by knockdown of Matrin3 (or PTB) knockdown. One striking difference was that the introns flanking Matrin3 repressed exons are on average 1 kb longer than introns flanking Matrin3-activated, PTB-repressed, PTB-activated or control exons (Fig4A).

We next looked for enrichment of pentamer sequence motifs associated with Matrin3-regulated exons, compared to control unregulated cassette exons, across seven transcript locations (cassette exons, flanking constitutive exons, 5′ and 3′ end of each flanking intron). Numerous motifs were enriched (FDR < 0.05) in the introns flanking Matrin3-repressed exons, but none within exons or in any location associated with Matrin3-activated exons (Fig4B, Supplementary Fig S4, Supplementary Table S4). Motifs associated with Matrin3-repressed exons were heterogeneous and included a number of pyrimidine motifs associated with PTB (e.g. TTCTT, TCTTT). The enrichment was also observed using a control set consisting of exons including PTB-regulated, Matrin3-independent exons. Most of the remaining motifs had high pyrimidine content, with one or two interrupting purines; more than half of the motifs immediately flanking Matrin3 repressed exons had a single purine. Individual analyses of RNA binding by Matrin3 have not revealed a clear consensus sequence (Hibino *et al*, 2006; Salton *et al*, 2011; Yamazaki *et al*, 2014). However, Matrin3 was one of 207 RBPs whose optimal sequence was determined by the RNA-compete array-based selection (Ray *et al*, 2013). We therefore used heptamer position frequency matrices to look for enrichment of RNA-compete motifs. Once again, enriched motifs were found only in the introns flanking Matrin3-repressed exons, and in no locations associated with Matrin3 activation (Fig4B, Supplementary Table S5). Strikingly, the Matrin3 motif was the only enriched RNA-compete motif upstream of repressed exons and was one of only four motifs on the immediate downstream side. Other enriched motifs included PTBP1, PCBP1, HuR and ZCRB1; this may be either due to a consequence of these proteins functioning as a co-regulator of Matrin3 or simply due to the similarity between the sequences of the binding sites. Taken together, the k-mer and RNA-compete motif enrichments suggest that Matrin3 might bind directly to the longer introns flanking repressed exons, but that activation by Matrin3 might be indirect.

### Matrin3 binds widely around repressed exons

**Figure 5:**
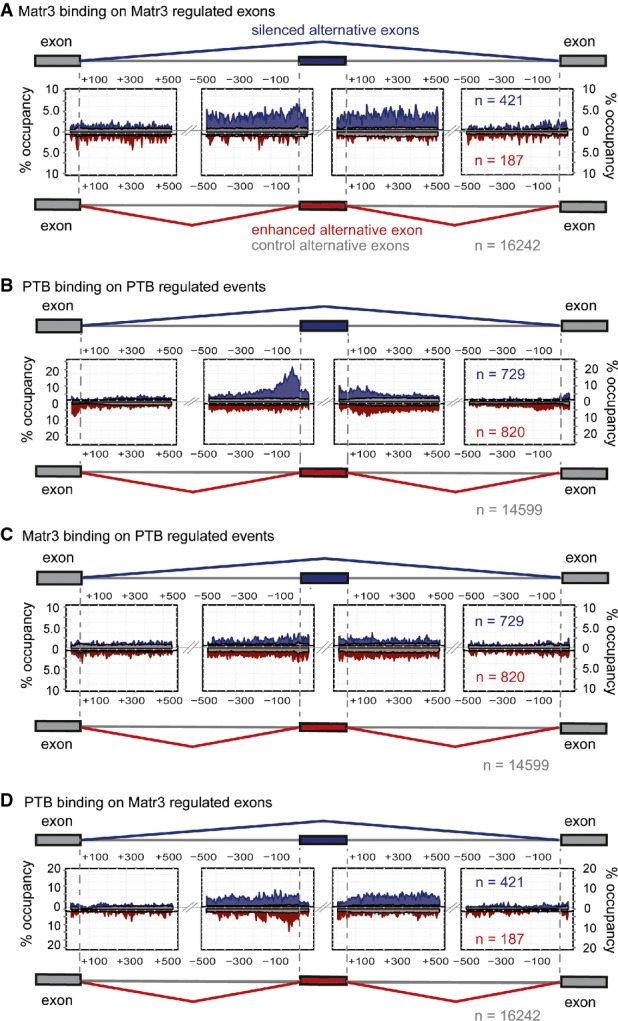
Matrin3 and PTB splicing maps A Matrin3 crosslinking in Matrin3-regulated pre-mRNAs where position of crosslinked nucleotides was mapped onto the regulated exon and the 500 nucleotides upstream and downstream of its 3′ss and 5′ss, respectively, the upstream flanking exon and 500 nucleotides downstream of its 5′ss and the downstream flanking exon with 500 nucleotides upstream of its 5′ss. The iCLIP tags were mapped onto silenced ASE (blue, *n* = 421), enhanced ASE (red, *n* = 187) and to control ASE (grey, *n* = 16,242), and percentage of occupancy is plotted.B Same as in (A) but using iCLIP tags obtained from PTB iCLIP and mapped onto PTB/nPTB-regulated ASE, silenced ASE (blue, *n* = 729), enhanced (red, *n* = 820) and control ASE (grey, *n* = 14,599).C Matrin3 crosslinked nucleotides mapped onto PTB-regulated ASE.D PTB crosslinked nucleotides mapped onto Matrin3-regulated ASE.

To directly address the relationship between Matrin3 splicing activity and RNA binding, we carried out crosslinking and immunoprecipitation (iCLIP) in HeLa cells (König *et al*, 2010). We obtained a total of 3,496,801 cDNA reads after collapsing PCR duplicates that mapped uniquely onto the genome. A Matrin3 splicing map was generated using the Matrin3 iCLIP binding microarray data sets (Fig5). Matrin3 binding was elevated in long intronic regions immediately flanking repressed exons (Fig5A, blue). In contrast, the flanking constitutive exons, their immediate intron flanks and all regions associated with Matrin-activated exons (Fig5A, red) showed binding levels only slightly elevated above control cassette exons (Fig 5A, grey). This differential observed binding agrees well with the motif enrichments (Fig4B, Supplementary Tables S4 and S5). In contrast to many other splicing regulators (Licatalosi & Darnell, 2010; Witten & Ule, 2011), Matrin3 binding was uniformly elevated within 500 nt of repressed exons, with no discrete peaks (Fig5A). This uniform binding was not simply a result of higher steady-state levels of these RNA regions, because TIA1 (Wang *et al*, 2010) and U2AF65 (Zarnack *et al*, 2013) iCLIP tags showed a similar density on control and Matrin3-regulated exons (Supplementary Fig S2A). The uniform binding was also observed around individual exons (e.g. ZMYND8 and ADAR1B, Supplementary Fig S2D) and so did not result from averaging across large numbers of introns with discreet peaks at different locations. We also noted that elevated Matrin3 binding extended into the repressed exons (Fig5A), even though no enriched motifs had been observed in this location (Fig4B). A possible explanation for the extended region of elevated binding including the exon is that binding initiates at high affinity specific motifs in the flanking introns and then extends by Matrin3 oligomerization with less specific RNA binding (see Discussion). We did not observe a clear correlation between tag density and degree of splicing change upon Matrin3 knockdown, which may be related to lack of saturation of the iCLIP library. Nevertheless, the density of Matrin3 tags around repressed compared to control exons was highly significant (*P* < 0.0001, χ^2^ test).

In view of our initial identification of Matrin3 as a PTB-interacting protein (Figs1 and 2), the small overlap of target splicing events was somewhat surprising (Fig3). We therefore explored the relationship of Matrin3 and PTB binding to each other's target RNAs. We carried out iCLIP for PTB in HeLa cells and used the previous HJAY data set for PTB/nPTB knockdown (Llorian *et al*, 2010). We obtained a total of 5,981,600 cDNA reads that mapped uniquely onto the genome. The resultant PTB splicing map showed the characteristic peak within 100 nt upstream of PTB-repressed exons, while PTB-activated exons showed highest levels of binding on the downstream side of activated exons and within the upstream constitutive exon (Fig5B) (Xue *et al*, 2009; Llorian *et al*, 2010). Mapping Matrin3 iCLIP tags onto PTB-regulated exons showed binding only slightly above control cassette exons (Fig5C). PTB had a higher level of occupancy upstream of Matrin3-activated exons, even though Matrin3 itself does not appear to bind in this location (Fig5A and D). It is possible that for PTB-repressed, Matrin3-activated exons, such as PTBP2 exon 10 and PTBP3 exon 2, Matrin3 could antagonize PTB activity without binding RNA. How Matrin3 would activate PTB-independent exons without binding RNA remains unclear. PTB iCLIP tags showed a marked enrichment across the ± 500 nt intron flanks of Matrin-repressed exons, very similar to the pattern of Matrin3 binding (Fig5D), despite the fact that 80% of these exons are PTB independent. Indeed, PTB iCLIP tags could be observed in individual introns flanking Matrin3-repressed, PTB-independent exons (e.g. ADAR1B, Fig2D). The lack of positional binding in vicinity to the 3′ splice site of Matrin3-repressed exons clearly distinguishes this non-functional mode of PTB binding from authentic PTB-regulated target sites (Fig5B and D). We thus exclude that this binding reflects co-regulated events. Instead, this strongly suggests that PTB is recruited by Matrin3 to the introns flanking Matrin3-repressed exons even if it does not contribute to exon repression.

### Matrin3 requires its RRM and PRI motif for splicing regulation

**Figure 6:**
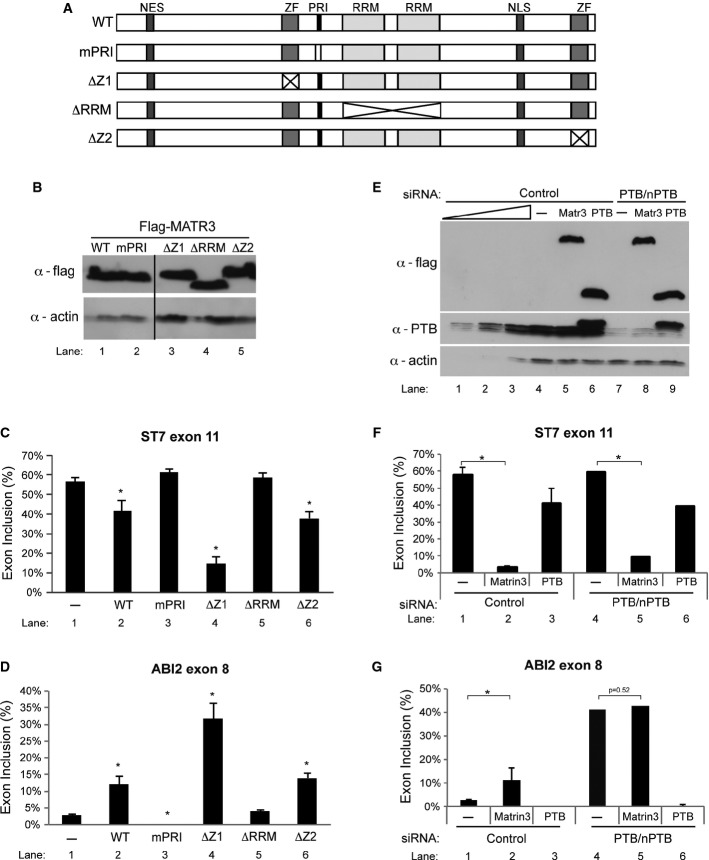
Matrin3 splicing function requires PRI and RRMs A Schematic representation of Matrin3 with its domains in the wild-type construct (WT) and the mutants used in overexpression, mPRI which has corresponding domain deleted in each one of the mutants, mPRI where the PRI motif is mutated from GILGPPP to GAAAPPA, ΔZ1 where the first zinc finger is deleted, ΔRRM where both the RRM domains are deleted and ΔZ2 where the second zinc finger is deleted.B Western blot of the overexpressed Matrin3 and its mutants probed with anti-FLAG and anti-actin antibodies.C, D RT–PCR analysis using primers specific to the ST7 exon 11 (C) and ABI2 exon 8 (D) splicing reporters, respectively, of samples where FLAG-tagged Matrin3 and its mutants have been overexpressed. Quantification of at least three replicates for each condition is shown as a histogram of the percentage of exon inclusion. *P < 0.01 compared with control sample (−).E Western blot of overexpressed FLAG-tagged Matrin3 and PTB, in control siRNA and PTB/nPTB siRNA-transfected cells, probed with anti-FLAG to detect overexpressed proteins, anti-PTB to detect the knockdown levels and anti-actin to confirm protein loading. A titration of control sample (lane 1—12.5%, lane 2—25%, lane 3—50% and lane 4—100%) is also included to assess the levels of knockdown. The band present above the PTB doublet in lane 6 is FLAG-tagged PTB.F, G RT–PCR analysis using primers specific to the ST7 exon 11 (F) and ABI2 exon 8 (G) splicing reporters, respectively, of samples where Matrin3 or PTB expression vectors were co-transfected, in control siRNA and PTB/nPTB siRNA-transfected cells. Quantification of at least three replicates for each condition is shown as a histogram of the percentage of exon inclusion. *P < 0.01 when compared with control sample in each condition (-). Source data are available online for this figure

In order to begin to understand the possible mechanisms by which Matrin3 regulates splicing, we designed two different splicing reporters based on ABI2 exon 8, which is activated by Matrin3 and repressed by PTB, and ST7 exon 11, which is Matrin3 repressed and PTB independent. The exons with flanking intronic regions were cloned into an exon-trapping vector with flanking constitutive exons and splice sites to generate a 3 exon construct. The splicing reporters were co-transfected into HeLa cells along with Matrin3 expression vectors with a range of domain deletions and mutations (Fig6A). All FLAG-tagged Matrin3 proteins were expressed at comparable levels (Fig6B), allowing direct comparison of their activities. The ABI2 exon 8 showed increased inclusion in response to Matrin3 co-transfection (Fig6D, lanes 1, 2). This activity was unaffected by deletion of zinc finger 2 and apparently increased by deletion of zinc finger 1 (Fig6D, lanes 4 and 6). In contrast, deletion of the RRMs impaired activity (Fig6D, lane 5). This shows that although Matrin3-activated exons showed no association with enriched motifs (Fig4B) or observed binding (Fig5), Matrin3 still requires its RRM domains for activity. Mutation of the PRI led to a complete loss of activity (Fig6D, lane 3), suggesting that the ability of Matrin3 to interact with PTB is important for its antagonistic activity upon this exon. The ST7 reporter alone showed ∽50% exon inclusion and, as expected, this level decreased upon overexpression of Matrin3 (Fig6C, lanes 1 and 2). The response to the various mutations was very similar to ABI2 exon 8. Deletion of the zinc finger domains (Fig6C, lanes 4 and 6) did not impair Matrin3 repressor activity, while deletion of the RRMs (Fig6C, lane 5) or mutation of the PRI (Fig6C, lane 3) abolished activity. The response to RRM mutation is consistent with the observed direct binding around Matrin3-repressed exons (Fig5). However, the effect of the PRI mutation is striking in view of the fact that ST7 exon 11 is independent of PTB (Fig3F). This suggests that other proteins might bind to this motif and be required to co-regulate events independent of PTB.

To address whether the effect of the Matrin3 PRI mutation is related to its interaction with PTB or other PRI-interacting proteins, we tested the effects of Matrin3 overexpression in combination with PTB knockdown upon the minigenes (Fig6E). The ST7 minigene, like the endogenous gene (Fig3F), was unresponsive to PTB/nPTB knockdown and responded to Matrin3 overexpression independently of PTB levels (Fig6F, lanes 1, 2, 4, 5). In contrast, the ABI2 minigene responded to both overexpression and knockdown of PTB (Fig6G, lanes 1, 3, 4, 6). Although Matrin3 overexpression promoted ABI2 exon 8 inclusion in the presence of PTB, upon PTB knockdown Matrin3 had no effect (Fig6G, compare lanes 1, 2 with 4, 5). This suggests that Matrin3 acts mainly to antagonize PTB when regulating ABI2 exon 8. In summary, the structure-function analysis indicates that Matrin3 requires its PRI motif and one or both RRM domains to act as a splicing regulator.

## Discussion

### Matrin3 is a splicing regulator

Matrin3 has long been suspected to play a role in RNA metabolism due to the presence of RNA-binding domains and its interactions with multiple other RNA-binding proteins, some with known roles in splicing regulation and other RNA processing roles (Polydorides *et al*, 2000; Zeitz *et al*, 2009; Salton *et al*, 2011). Nevertheless, evidence for a direct functional role of Matrin3 in cellular mRNA metabolism has been missing. Using a combination of RNAi knockdown, transcriptome profiling and iCLIP, we show unequivocally that Matrin3 is a strong regulator of multiple splicing events. Knockdown of HeLa cell Matrin3 leads to dysregulation of 667 ASEs, while iCLIP shows that Matrin3 binds directly to the introns flanking repressed exons. Analysis of functional terms associated with the regulated cassette exons revealed enrichment of genes encoding chromatin/chromatin-binding proteins and cytoskeletal proteins (Fig3C). Matrin3 has a number of previously reported roles including involvement in DNA damage response (Salton *et al*, 2010), and as a co-factor of Rev-mediated export of HIV1 RNA (Kula *et al*, 2011, 2012; Yedavalli & Jeang, 2011). The only other suggested widespread RNA-related function for Matrin3 is mRNA stabilization (Salton *et al*, 2011). Among the relatively few transcript level changes that we observed upon Matrin3 knockdown, we were able to identify nine of the 77 previously reported down-regulated mRNAs (Salton *et al*, 2011). Notably in five of the nine mRNAs, we observed ASEs that shifted upon Matrin3 knockdown towards isoforms with premature termination codons (PTCs) that would be expected to lead to nonsense-mediated decay (NMD). For example, in *THUMPD2* pre-mRNA, Matrin3 represses splicing of the PTC-containing exon 7, while in *ACAD9* upon Matrin3 knockdown, 5′ splice site selection on exon 1 shifts to an alternative downstream site which leads to inclusion of a PTC. Indeed, of the 18 Matrin3-repressed cassette exons in genes whose expression is down-regulated more than 1.5-fold, 13 are predicted to lead to NMD upon exon inclusion and hence to be destabilized upon Matrin3 depletion. Likewise, two of the four Matrin3-activated exons in genes that are more than 1.5-fold down-regulated upon Matrin3 knockdown cause NMD upon skipping, including the exon 10 of nPTB (Fig3F). While we cannot rule out Matrin3 acting directly to stabilize some mRNAs, our data suggest that at least in some cases alterations of mRNA levels are attributable to Matrin3 action as a splicing regulator of ASEs which lead to NMD. Another possible indirect influence of Matrin3 upon mRNA levels is via its regulation ASEs in chromatin-related proteins (Fig3C).

The characterization of Matrin3 as a splicing regulator is relevant for interpreting the basis of Matrin3-associated pathologies, such as ALS (Johnson *et al*, 2014) and distal myopathies (Senderek *et al*, 2009). Like many other RNA-binding proteins associated with neurodegenerative diseases, Matrin3 has extensive low-complexity disordered segments. Mutations in these regions of other proteins, such as TDP43, can lead to intracellular insoluble protein inclusions which can be directly proteotoxic or can lead to dysregulated RNA metabolism (Buratti & Baralle, 2012). Likewise, Matrin3 mutations might directly affect its splicing regulatory activity or reduce its effective concentration, either of which could have consequences for target ASEs. Notably, nuclear and cytoplasmic localization of isoforms of dystrophin (Gonzalez *et al*, 2000), the protein primarily affected in Duchenne's and Becker's muscular dystrophies, is regulated by AS of exon 78. Both Matrin3 and PTB repress inclusion of this exon (Fig3F).

### Mechanism of Matrin regulation of splicing

Matrin3 function as a direct splicing repressor is supported by its observed binding around repressed exons (Fig5) along with the enrichment in flanking intron segments of optimal binding motifs for its RRM motifs (Fig4B). Consistent with this, it requires intact RNA-binding domains, but not DNA-binding domains, for its splicing repressor activity (Fig6). Nevertheless, further evidence of its direct mode of action could be provided by *in vitro* analyses of its binding to regulated RNAs, demonstrating that specific binding sites are required for its splicing repressor activity, and more detailed analysis of the roles of the individual RRM domains. Unexpectedly, deletion of the ZF1 DNA-binding domain actually enhanced the activity of transfected Matrin3 (Fig6). One possible explanation for this observation is that deletion of ZF1 affects the distribution of Matrin3 between pools that are active for splicing regulation or that are localized elsewhere.

The majority of Matrin3 target exons are unaffected by PTB (Fig3), even though we initially identified Matrin3 via its interaction with PTB RRM2, part of the minimal repressor domain of PTB (Figs1 and 2) (Robinson & Smith, 2006). Indeed, we observed enhanced recruitment of PTB around Matrin3-repressed exons and, importantly, the distribution of PTB resembled the broad distribution of Matrin3 around its own targets (Fig5A and D) and lacked the distinctive peak of PTB binding observed upstream of PTB-repressed exons (Fig5B). This suggests that PTB can be recruited to Matrin3-regulated exons, even when it is not functionally required, as in the ST7 exon 11 (Figs3F and 6). However, we observed that repression of ST7 exon 11 depended absolutely upon the Matrin3 PRI motif (Fig6), which suggests that Matrin3 repressor activity at this exon might require interaction with proteins other than PTB via its PRI. The binding of Matrin3 across extensive regions flanking and within repressed exons (Fig5A) is suggestive of a mechanism of initial binding to specific sites followed by spreading. This is consistent with the observed enrichment of k-mers and the RNA-compete motif exclusively in the immediate 250 nucleotides of the flanking introns (Fig4), and with its reported propensity for self-association (Zeitz *et al*, 2009). Similar models were originally suggested for repression by PTB (Wagner & Garcia-Blanco, 2001) and hnRNPA1 (Zhu *et al*, 2001), but analysis of numerous model systems has shown that this is not a common mechanism for these proteins (e.g. Cherny *et al*, 2010), and their splicing maps show distinct peaks of enriched binding (Xue *et al*, 2009; Llorian *et al*, 2010; Huelga *et al*, 2012). In contrast, the Matrin3 splicing map (Fig5A) is consistent with a general mechanism of action in which initial binding of Matrin3 at specific sites is followed by propagative binding across a wide region of RNA, leading to repression of the targeted exon. This model is amenable to testing by various methods including single molecule analysis (Cherny *et al*, 2010).

How Matrin3 might promote exon inclusion is less clear. Splicing maps indicate that binding around activated exons is not much above background (Fig5A) and no motifs were enriched (Fig4). Nevertheless, in the ABI2 minigene assay, the RRMs were required, along with the PRI motif (Fig6). One explanation could be that activated exons are mainly indirect targets, and changes in their splicing result from the primary actions of Matrin3, which require RNA binding. Another possibility, in cases where Matrin3 activation directly opposes PTB repression, is that interaction with Matrin3 might antagonize PTB activity. Although Matrin3 binding is low around Matrin3-activated exons (Fig5A), PTB binding shows an upstream peak (Fig5B) consistent with PTB repression. A notable example of this is the Matrin3-activated nPTB exon 10 (Fig3F), a well-known target of PTB repression (Boutz *et al*, 2007; Makeyev *et al*, 2007; Spellman *et al*, 2007).

### PRI-protein interactions

In addition to Matrin3 and Raver1, we found a number of other interesting factors that bound to PTB RRM2 and were sensitive to the Y247Q mutation (Joshi *et al*, 2011), suggesting that their interactions might also be mediated by PRI motifs (Fig1). These included splicing regulators, proteins involved in the co-transcriptional-dependent regulation of splicing and 3′ end processing factors. We did not identify a number of previously identified PTB interactors, including hnRNP-L, PSF, hnRNPA1, hnRNPA2B1, hnRNPC and MRG15 (Patton *et al*, 1993; Hahm *et al*, 1998; Luco *et al*, 2010; King *et al*, 2014), although we did identify the helicase DDX3X (King *et al*, 2014). The lack of overlap could be because we focused on proteins that interact primarily via RRM2. Interesting novel PTB interactors with possible relevance for PTB's splicing regulatory activities include KIAA1967/DBC1/CCAR2 and its paralogue CCAR1, both of which bound strongly to PTB RRM2 in a manner that was sensitive to the PTB Y247Q mutation (data not shown). CCAR1 is a spliceosomal A-complex component that interacts with U2AF_65_ (Hegele *et al*, 2012). DBC1 together with ZNF326/ZIRD forms the DBIRD complex, which integrates alternative splicing with RNA polymerase II transcription (Close *et al*, 2012). This complex is required for RNA Pol II to efficiently transcribe AT-rich regions upstream of some exons. Depletion of the DBIRD complex, by DBC1 or ZIRD knockdown, leads to accumulation of stalled RNA Pol II in these AT-rich regions, which enhances the inclusion of the proximal exon (Close *et al*, 2012). It will be interesting to determine whether interaction of PTB with DBC1 occurs as part of the DBIRD complex, as a distinct DBC1-PTB regulatory axis, or possibly in coordination with chromatin association PTB-MRG15 interaction (Luco *et al*, 2010). DBC1 and CCAR1 both have potential N-terminal PRI motif, and DBC1 has an additional one. These motifs are conserved across multiple vertebrate species, but we have yet to determine whether they are functional. While we have identified a number of potentially interesting PTB-interacting proteins, it is also likely that we have failed to identify other interacting proteins for which the RRM2 interface is either not sufficient or not necessary. Notably, Matrin3 interacted more strongly with full-length PTB than RRM2 alone, while Raver1 had the opposite response (Fig1D). This suggests that identification of the full range of proteins that interact with PTB will require additional analyses using full-length PTB and possibly its PTB1 and 4 spliced isoforms (Wollerton *et al*, 2001).

While the Matrin3 PRI matches the consensus [S/G][I/L]LGxϕP based on mutagenesis of Raver1 motifs, it is notable that it shows absolute conservation (GILGPPP) across all available vertebrate species, whereas the two main Raver1 motifs show variation at positions 5 and 6. This suggests that while Raver1 interacts only with PTB through its PRI, Matrin3 might bind also to other RRM containing proteins and is therefore subject to greater sequence constraints. Consistent with this, we observe that activity of Matrin3 on ST7 splicing reporter requires the PRI motif (Fig6C), even though this event is PTB independent (Fig3F). Comparison of proteins co-immunoprecipitated with wild-type and PRI mutant Matrin3 showed only two obvious differences, which mass spectrometry revealed to be both isoforms of PTB (Supplementary Fig S5). Further investigation will be carried in the future to determine the additional PRI-interacting factors for Matrin3 activity. Several proteins from previous Matrin3 interactome studies contain RRM domains and might be candidate PRI-interacting proteins, including RBMX, SAFB, HNRNPL, U1SNRNBP, SFRS7, SLTM, PABPC1, PSF and TDP43 (Zhang & Carmichael, 2001; Zeitz *et al*, 2009; Salton *et al*, 2011; Johnson *et al*, 2014). Additional candidates suggested by association of their RNA-compete motifs (Ray *et al*, 2013) with Matrin3-repressed exons are HuR and ZCRB1 (Fig4). Strikingly, the strongest interaction seen in yeast two-hybrid screens was Matrin3 binding to itself (Zeitz *et al*, 2009), raising the possibility that its PRI could interact with an RRM domain of another Matrin3 monomer. We tested this hypothesis and saw no binding between the PRI and RRM domains of Matrin3 (data not shown). A further possibility is that the Matrin3 PRI interacts with domains other than RRMs, a possibility which could be addressed by unbiased interaction screening of wild-type and PRI mutant Matrin3 by quantitative proteomics.

In summary, we have established a clear role for the nuclear matrix protein Matrin3 in directly regulating a network of alternative splicing events, a small proportion of which are also co-regulated by PTB. This insight should assist future investigations into the cellular roles of Matrin3, as well as the basis of molecular pathologies associated with Matrin3 mutations (Senderek *et al*, 2009; Johnson *et al*, 2014).

## Materials and Methods

### Tissue culture, DNA and siRNA transfection and analysis

HeLa S3 and HEK-293T cell lines were cultured using standard procedures. siRNA-mediated knockdown was carried out by doing a 2-hit transfection of control, Matrin3 or PTB targeting siRNAs. Cells were plated in a 35-mm well in day 1, followed by transfection of 40 pmol of siRNA (40 pmol of control siRNA, 40 pmol of Matrin3 siRNA, and for PTB/nPTB double-knockdown 20 pmol of PTB siRNA and 20 pmol of nPTB siRNA) in day 2 and day 3, using Oligofectamine (Invitrogen) and Lipofectamine (Invitrogen) in each day, respectively, with cells being harvested on day 5. siRNAs were purchased from Dharmacon, and mRNA targets for gene-specific knockdown were (5′-3′) Matrin3 M3 AAAGACUUCCAUGGACUCUUA (Salton *et al*, 2010), PTB P1 AACUUCCAUCAUUCCAGAGAA, nPTB N1 AAGAGAGGAUCUGACGAACUA and control C2 AAGGUCCGGCUCCCCCAAAUG (Spellman *et al*, 2007). PTB knockdown was carried out by transfecting siRNAs targeting both PTB (P1) and its neuronal paralogue (N1), nPTB, which otherwise partially compensates for PTB (Spellman *et al*, 2007). Knockdown efficiency was monitored by lysing a 35-mm well for each condition with protein loading buffer directly, followed by SDS–PAGE and Western blot using anti-Matrin3 (sc-55723; Santa Cruz), anti-PTB (Spellman *et al*, 2007) and anti-actin antibodies (A2066; Sigma). Other antibodies used were FLAG M2 monoclonal (Sigma) and Raver1 rabbit polyclonal against the RRM domains. For immunoprecipitation, cells were transfected using Lipofectamine 2000 (Invitrogen) and harvested 48 h post-transfection in 200 μl of lysis buffer (Huttelmaier *et al*, 2001), 5% of cell extract was analysed by Western blot to check for expression of transfected proteins, and the remainder was used for immunoprecipitation using 5 μl of anti-FLAG antibody (Sigma M2) with 10 μl of protein G sepharose, previously blocked with 2% BSA. Antibody–protein complex was allowed to bind for 2 h at 4°C, followed by extensive washes, and eluted in 30 μl of protein loading buffer. The entire sample was subjected to Western blot and probed against PTB. For splicing reporter analysis, we transfected cells in triplicates with 2 μg of effector DNA encoding Matrin3 and its mutants together with 200 ng of the indicated splicing reporter. Forty-eight hours post-transfection cells were harvested using TRI reagent (Sigma) and 2 μg of total RNA was used for reverse transcription using either splicing reporter-specific oligo, or in the case of validation of microarray ASE, oligo dT, using superscript II (Invitrogen). For the splicing reporters, the splicing pattern was analysed by PCR using the primers GFPN and CG3 as described previously (Llorian *et al*, 2010). For endogenous ASE analysis, gene-specific primers were used (Supplementary Materials and Methods). All PCRs were carried out using the Jumpstart Taq polymerase (Sigma), and the products were separated and quantified on a QIAXcel capillary electrophoresis system (Qiagen).

### DNA constructs

GST-PTB1 and GST-SXL used for pull-down of PRI motifs have been described previously (Rideau *et al*, 2006). GST-RRM2 and GST-RRM2 Y247Q were PCR-amplified and cloned into the EcoRI site in pGEX3 as described (Joshi *et al*, 2011). To generate the FLAG-Matrin3 PRI-MS2, we cloned into the AvrII and MluI sites pre-annealed oligos encoding the indicated protein sequence. FLAG-MS2, FLAG-Raver1 full-length wild-type and mutant, FLAG-Raver1 PRI have all been described previously (Rideau *et al*, 2006). Full-length Matrin3 was PCR-amplified from human cDNA and cloned into the MluI sites of a pCI-FLAG vector (Promega). Deletion mutants were generated by divergent PCR and mPRI by site-directed mutagenesis (Promega). The splicing reporters were constructed by PCR amplifying the regulated exon and the flanking region and cloning them into the Asp718I and EcoRV sites of a GFP expression cassette where an intron has been inserted into the second codon. The cloning sites are localized in the middle of this intron so will generate three exon splicing reporters upon cloning (Wollerton *et al*, 2004). All DNA constructs were confirmed by sequencing.

### GST expression, purification and pull-down

Pull-down of nuclear proteins using GST-RRM2 and GST-RRM2 Y247Q was carried out as follows: 2 μg of purified recombinant protein was pre-bound to glutathione sepharose 4B beads for 1 h. To each GST protein, 300 μl of HeLa nuclear extract was added, together with RNase A to a final concentration of 5 μg/ml. The mixture was incubated for 2 h at 4°C rotating, followed by extensive washes and elution using 30 μl of protein loading buffer. Five microlitre was used for silverstain analysis, and the remainder of the pull-down was run on a SDS–PAGE followed by mass spectrometry of the entire section of the lane above the recombinant protein (see Fig1A). Expression and purification of GST-RRM proteins has been described elsewhere (Joshi *et al*, 2011). Pull-downs of *in vitro* translated protein by GST-PTB were carried out as described (Rideau *et al*, 2006) using GST-SXL as negative controls. GST-SXL protein was produced from the plasmid pGEX CS NR SXL XW II, which was a kind gift from J. Valcárcel, Centre de Regulació Genòmica. RNase treatment of pull-downs was carried out in two steps: the *in vitro* translations were terminated with a 15-min incubation at 30°C with 25 μg/ml RNase A, and RNase A was added at 0.5 μg/ml to the pull-down pre-incubation for 3 h at 4°C.

### Human Junction microarray experiments and validation

RNA from four biological replicates of control or Matrin3 siRNA-transfected HeLa cells was isolated using TRI reagent (Life Technologies). Total RNA was hybridized to Human Affymetrix Exon-Junction Array (HJAY) by Genecore (EMBL). The microarray data were analysed using ASPIRE (Analysis of SPlicing Isoform REciprocity) 3.0 (König *et al*, 2010; Wang *et al*, 2010). It analyses signal in reciprocal probe sets to monitor changes in alternative splicing events, from which we applied a threshold of |dIrank| ≥ 1 and obtained 667 alternative splicing events (Supplementary Table S3). Matrin3, PTB/nPTB and triple Matrin3/PTB/nPTB were carried out and cDNA was produced from extracted RNA using oligo dT. The splicing pattern of each ASE was analysed by RT–PCR (for primer sequence, see Supplementary Materials and Methods).

### Bioinformatic analysis of Matrin3 regulated ASE

Analysis of enriched functional gene categories was carried out using PANTHER (Mi *et al*, 2013). Analysis of Matr3 intron length was done using ASPIRE [CS1] annotation in R version 3.0.1 and the ggplot2 and pgirmess packages. Since some alternative splice events have 0-length introns, intron length was analysed for alternative cassette exons only. For statistic analysis, data were tested with Kruskal–Wallis rank-sum test before doing multiple comparison testing. Motif enrichments were calculated using 100 bp of the flanking exons and the complete sequence of the cassette exons. For introns, we used maximum intronic flanks of 250 nt, removing SS context to avoid BP, SS and PPT signals, 9 nt at donor side and 30 nt at the acceptor side (Bland *et al*, 2010), retrieving introns with a minimum length of 60 nt. We assess the enrichment of 5-mers and RNA-compete motif matches (Ray *et al*, 2013) with the following procedure implemented in a custom PHP script: Given a sample set *S* of *N* sequences and a control set *S*^(0)^ of *N*^(0)^ sequences, the number of times *n*_*a,i*_ that each motif *a* appeared in each sequence *i* was calculated. Likewise, for the control set, the number of occurrences 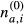 of each motif *a* per sequence *i* was also calculated. The expected density 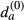 of each motif was also calculated as the ratio between the total number of occurrences in the control set over the total sequence length of the control set: 
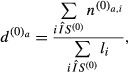
where *l*_*i*_ is the length of each sequence in the control set. For each sequence *i* in the sample set each motif *a*, it was recorded whether the observed motif count (*n*_*a,i*_) is greater than the calculated expected count (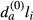): 
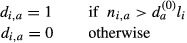


Similarly, for the counts in the control set: 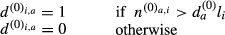


The sum of the *d*_*i,a*_ values over the sequences *i* represents the number of sequences for which the motif *a* has an observed count greater than expected. Thus, for each motif, the odds ratio (motif score) and corresponding *P*-value were the motif *a* has an obtained by performing a Fisher's test (one-tailed) with these sums counts for the sample set and the control set:

**Table:** 

5-mer *a*	More than expected	Less than expected
*S*	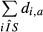	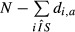
*S* ^(0)^	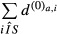	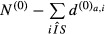

Statistically over-represented motifs were selected based on the Benjamini's and Hochberg's false discovery rate multiple test-corrected *P*-value (BH-FDR < 0.05). Statistical tests were performed and graphics were generated with R Development Core Team (2011). Additional scripts were written in PHP and Awk, and sequence logos were generated with seqlogo (Crooks *et al*, 2004).

### CLIP and splicing maps

iCLIP experiments were performed for Matr3 and PTB using antibodies targeting the endogenous protein, GTX47279 (GeneTex) and polyclonal anti-PTB serum (Spellman *et al*, 2007). Exponentially growing HeLa cells were washed once with PBS and cross-linked at 0.15 mJ/cm^2^ with a Stratalinker 2400 equipped with 254 nm light bulbs. Retrieval of protein-bound RNAs and preparation of Illumina-compatible DNA libraries were done as described in Huppertz *et al* (2014). To compute RNAmaps of Matrin3 and PTB binding on exon–intron boundaries, we assessed the positioning of cross-link sites. Cross-linked nucleotides are defined as the nucleotide upstream of mapped iCLIP cDNA tags as described before (König *et al*, 2010). For each position within the RNAmap, the number of cross-link nucleotides was counted as 1 if one or more cDNA tags matched the position, and then summed across all splice events. The summed cross-link count was divided by the number of splice events and plotted in 10 nucleotide bins. Thus, the resulting occurrence value reflects the number of exons with cross-linked nucleotides within the 10-nt window.

### Accession numbers

E-MTAB-3092, E-MTAB-3107, E-MTAB-3108.

## Supporting Information

Supplementary Table S5

Review Process File
