# Supplementary material for: Nuclear matrix protein Matrin3 regulates alternative splicing and forms overlapping regulatory networks with PTB
Source: EMBO J. 2015 Jan 19;34(5):653–68. doi: 10.15252/embj.201489852 (PMC4365034; doi:10.15252/embj.201489852)
Supplement: Supplementary file 1 [file embj0034-0653-sd1.pdf]

Overlapping Splicing Regulatory Networks of the Nuclear Matrix Protein Matrin3 and PTB

[1]Miguel B. Coelho, [2,3]Jan Attig, [4,5,6]Nicolás Bellora, [3,7]Julian König, [1,8]Martina Hallegger, [3]Melis Kayikci, [4,5]Eduardo Eyras, [2]Jernej Ule, [1]Christopher W.J. Smith*

[1]Department of Biochemistry, University of Cambridge, Tennis Court Road, Cambridge, CB2 1QW, UK

[2]Department of Molecular Neuroscience, UCL Institute of Neurology, Queen Square, London, WC1N 3BG, UK

[3]MRC-Laboratory of Molecular Biology, Hills Road, Cambridge CB2 0QH, UK

[4]Computational Genomics, Universitat Pompeu Fabra, Barcelona, Spain

[5]Catalan Institute for Research and Advanced Studies (ICREA), Barcelona, Spain

Current addresses:

[6]INIBIOMA, CONICET-UNComahue, Bariloche, Argentina,

[7]Institute of Molecular Biology gGmbH (IMB), Ackermannweg 4, 55128 Mainz, Germany

[8]Department of Physiology, Anatomy and Genetics, University of Oxford, Oxford, UK

*Corresponding author: cwjs1@cam.ac.uk

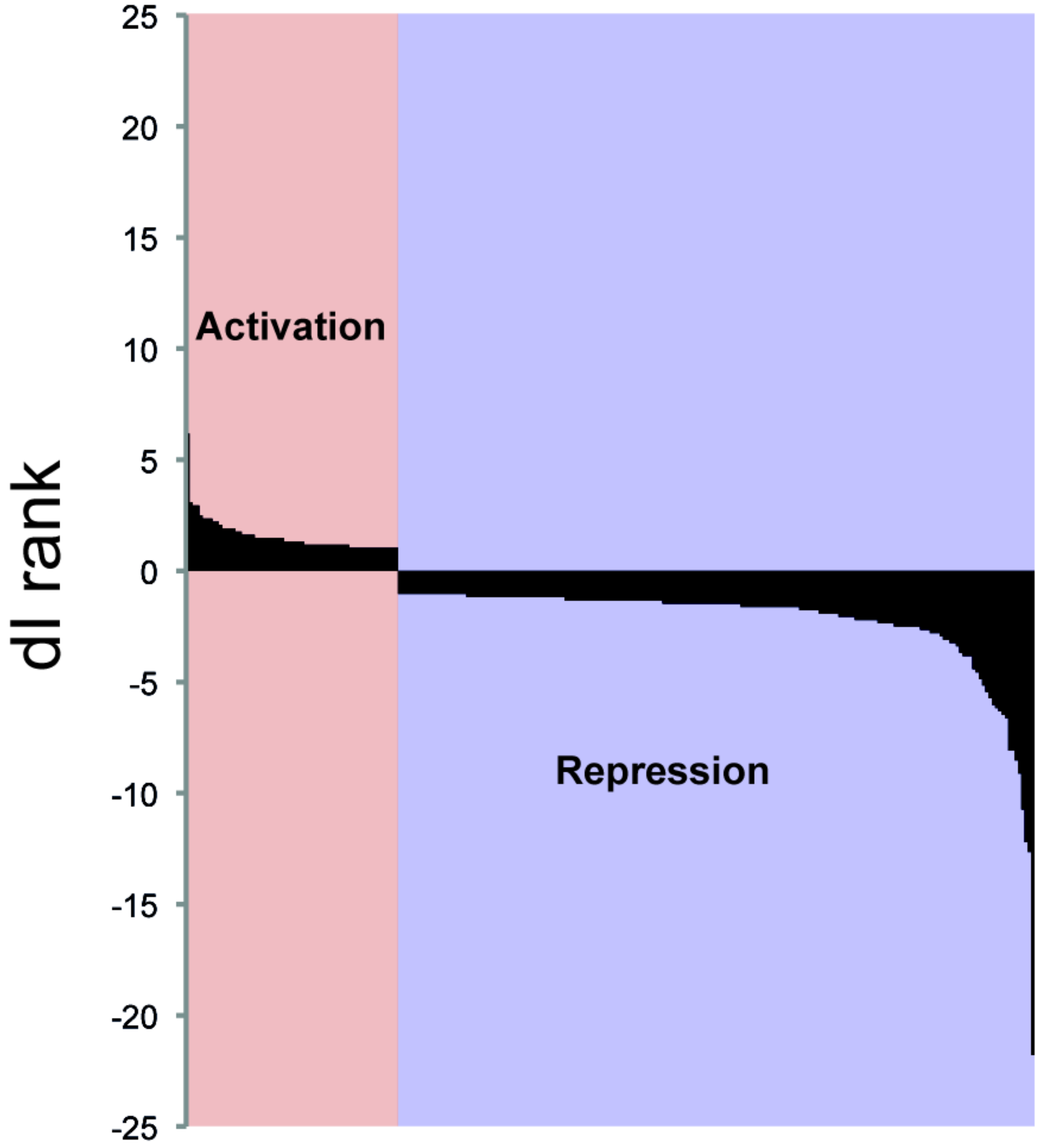

Supplementary Figure S1. Distribution of dIrank values for Matrin3 regulated cassette exons. Each bar of the histogram represents the dI rank of each regulated cassette exon.

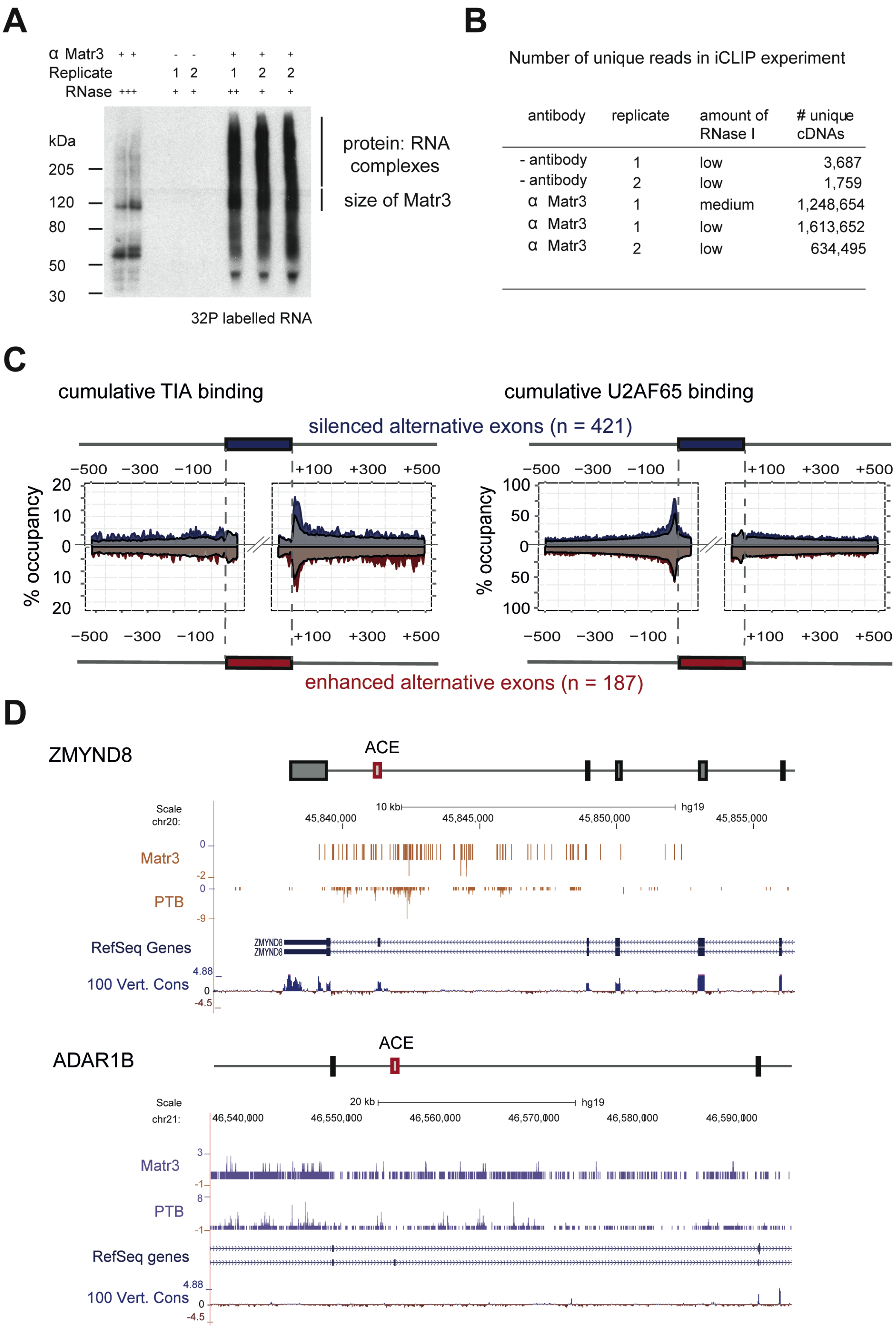

Number of unique reads in iCLIP experiment

antibody	replicate	amount of RNase I	# unique cDNAs
- antibody	1	low	3,687
- antibody	2	low	1,759
α Matr3	1	medium	1,248,654
α Matr3	1	low	1,613,652
α Matr3	2	low	634,495

Supplementary Figure S2 iCLIP experiments. (A) Analysis of crosslinked Matrin3 – RNA using denaturing gel electrophoresis. Different concentration of RNase were used +++ - , ++ - and + - . (B) Number of unique reads in the Matrin3 iCLIP experiment. (C) TIA1 (left) and U2AF65 (right) iCLIP tags mapped onto Matrin3 repressed (blue), activated (red), and unregulated control exons (gray). (D) Matrin3 and PTB iCLIP tags associated with two alternative cassette exons (ACE). ZMYND8 is repressed by Matrin3 and activated by PTB, while ADAR1B is repressed by Matrin3 and unaffected by PTB.

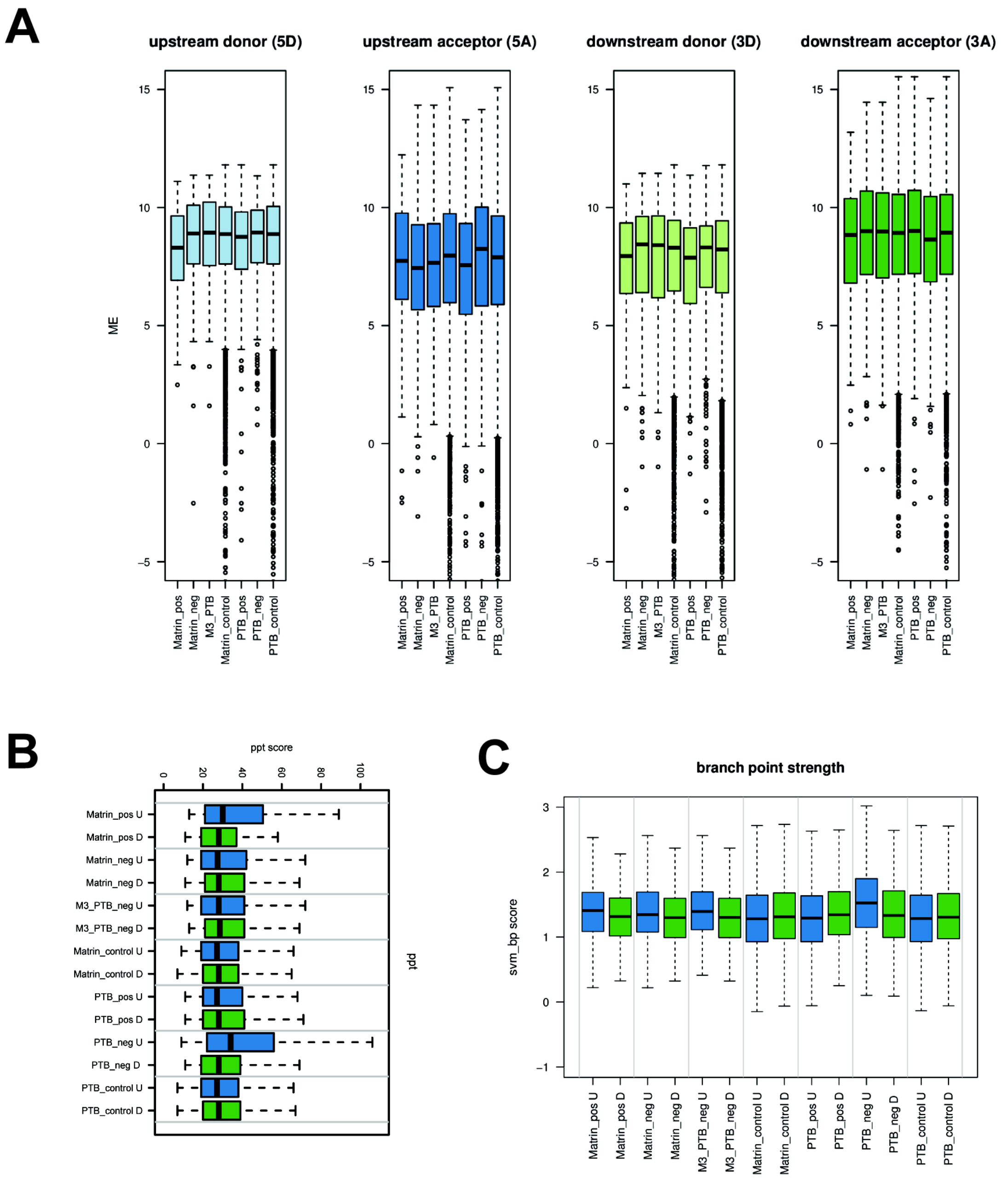

Supplementary Figure S3 Splice site, polypyrimidine tract and branchpoint strength of Matrin3 and PTB regulated ASE. (A) The splice site strength of the upstream 5' splice site (5D) and 3' splice site (5A), and downstream 5' splice site (3D) and 3' splice site (3A) were scored with Maximum Entropy. (B,C) Polypyrimidine Tract and Branch points of upstream (U, blue) and downstream (D, green) introns flanking Matrin3, PTB regulated and control ASE were scored using svm_bp (Corvelo et al, 2010).

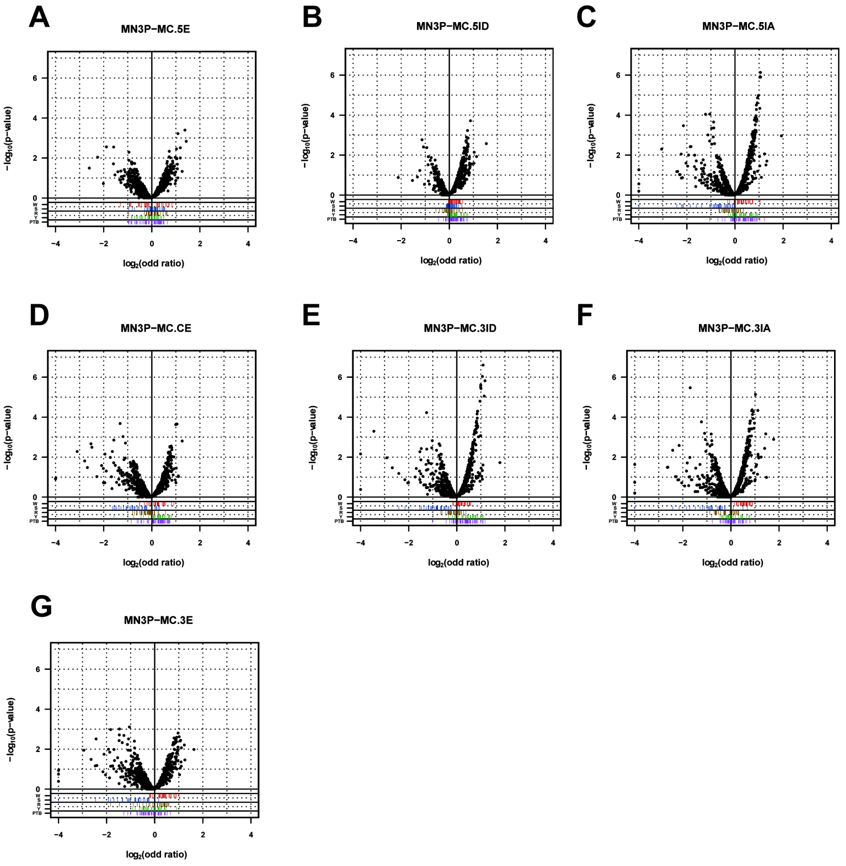

Supplementary Figure S4 Volcano Plots of Enriched 5-mer Motifs in Matrin3 Regulated ASE. The motifs were analysed along the regulated cassette exons (D), and the flanking upstream (C) and downstream (E) proximal intron, as well as the upstream exon (A) and its flanking downstream intron (B) and the downstream exon (G) and its upstream flanking upstream intron (F). In the panel below is shown the motifs that fit the following categories: CG-motifs (S, Red), AT-motifs (W, Blue), pyrimidine (Y, Brown), purine (R, Green) or PTB (P, Purple). The indicated motifs have 5 nucleotides of the corresponding category; for PTB we used the published PTB binding motifs YCTY or YTCY (Llorian et al, 2010).

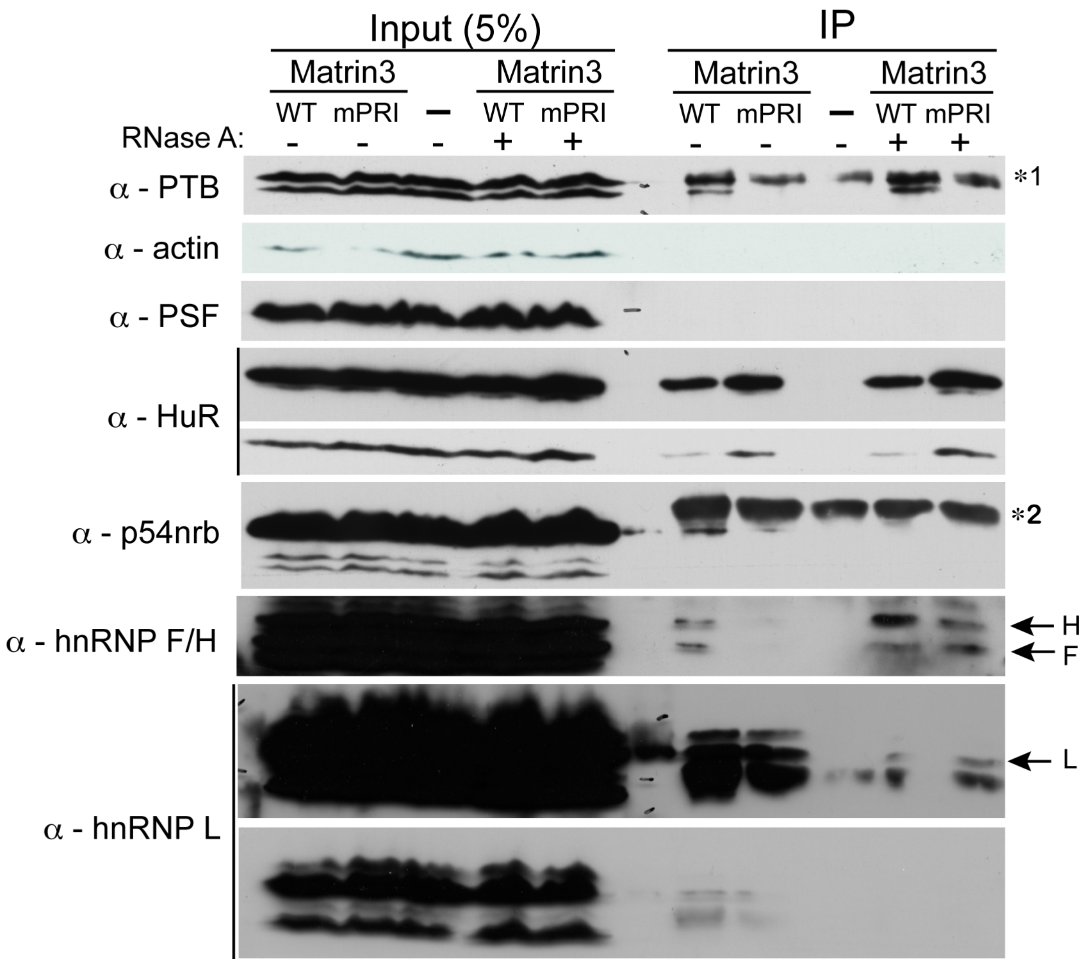

Supplementary Figure S5 Matrin3 IP of RRM-Containing Proteins. Several RRM containing proteins were probed by western blot of immunoprecipitated (IP) Matrin3 wild-type and mPRI mutant in presence and absence of RNase treatment. 5% of input sample is also shown on the left side of the panel and the IP on the right side. *1 – Signal due to cross reactivity with immunoglobins from the anti-flag antibody used for the IP overlaps with PTB4 isoform. *2 – Immunoglobin cross reactivity. Arrows indicates corresponding to indicated proteins.

Supplementary Materials and Methods

Primer sequences

Primer name	Sequence (5’-3’)	Purpose
Matr3_PRIa	CTAGGCAGTCTACAAATCCAGCACCAGGAATTCTGGGACCTCCACCTCCTTCATTTCATCTTGGGA	To generate Matrin3 PRI MS2
Matr3_PRIb	CGCGTCCCAAGATGAAATGAAGGAGGTGGAGGTCCCAGAATTCCTGGTGCTGGATTTGTAGACTGC	
Matr3_PRIa_mut	CTAGGCAGTCTACAAATCCAGCACCAGGAATTGCGGGACCTCCACCTCCTTCATTTCATCTTGGGA	To generate Matrin3 PRI MS2 with mutation L->A in PRI sequence
Matr3_PRIb_mut	CGCGTCCCAAGATGAAATGAAGGAGGTGGAGGTCCCGCAATTCCTGGTGCTGGATTTGTAGACTGC	
Matr3f	ATGTCCAAGTCATTCCAGCAGTCATCTCTCAGTAGGG	To amplify Matrin3 from human cDNA
Matr3r	AGTTTCCTTCTTCTGTCTGCGTTCTTCTGCC	
Matrin3dZnF1f	GGGATAACCCTTCGGTAAGAG	Used for deletion of ZF1
Matrin3dZnF1r	CTTCTTCTTGAAATCTACCCAGAATGG	
Matrin3dRRMf	GCTAGTTTCCACTCTGCC	Used for deletion of RRM 1 and 2
Matrin3dRRMr	AAACTGGTtCTGAGGATTCCaAACAGAGGC	
Matrin3dZnF2f	AGGTATCACATAGTCTATACCAACAGG	Used for deletion of ZF2
Matrin3dZnF2r	TTAAAGAAATTTCTGAATAAATTGGCAGAAGAACGCAGACAG	
ZMYND8e22f	TTTCTGGCTCCAGAGAGACG	Used to analyse the splicing pattern of the indicated ASE
ZMYND8e24r	TCTTCGTGCTGGTACTGGTG	
VEZTe10f	ACAGGATCTGGGACACACAG	
VEZTe12r	CCAACTTTTGATGGTTTCAGC	
C3orf17e1f	CGTAGCTCCGCCTTTCGTA	
C3orf17e3r	CTTGAATTGAGCCCTCCAAA	
DMDe77f	GCACAGGGTTAGAGGAGGTG	
DMDe79r	GCGGGAATCAGGAGTTGTAA	
VWA5Ae1f	AGCTGTTTTCACTCCGCTGT	
VWA5Ae3r	GAGGGTGAGTAGGCCACAGA	
TCF12e17f	CCTTCATCCCCAAGCTATGA	
TCF12e19r	TGGGATGGTCCCAATAAACT	
ST7e10f	ATGCAGAAAGCCTGGAGAGA	
ST7e12r	CCTTCAGGGCCTGCTTAAAT	
ACSL3e2	GGCGCATATCTTCAAAGCAC	
ACSL3e4	TGATGGTATGTTTTAGCTTCATGG	
PLEKHA3e3	CGTTGGTTTGTTTTAGATAATGGA	
PLEKHA3e5	AGCCACCTCTGTCTTTCAGC	
PIGXe2	GCATAAGGGCCATGTGTTCT	
PIGXe4	GCAAGTCCTCAATGCTTTCC	
ST7kpnIf	GGTACCAGTGCCACCCCTCTTTTCTT	Used to generate ST7 minigene
ST7ecoRVr	GATATCCGCATGAGTTTGGGATAGAA	
ABI2int7fAsp	AAGGTACCAAATCTGCTTATTTGATTCGAGGAG	Used to generate ABI2 minigene
ABI2int8rRV	AAGATATCTTCTGCATAAATTACCCAGTCTCAGG	

Bioinformatic analysis of Matrin3 regulated ASE

Intronic and exonic regions were extracted using custom scripts from the human assembly (GRCh37/hg19) downloaded from UCSC. The sets of regulated and control exons were defined after the analysis of Human Junction microarrays (HJAY) for each experiment. The regions used were the flanking regions of the upstream and downstream introns of the splicing event relative to the cassette exons. We discarded introns without 5' GT and 3' AG termination dimers. For the splice site strength analysis we extracted the intronic-exonic boundaries: 20-3nts at the acceptor site, and 3-6nts (exonic-intronic) at donors. In order to test the strengths of the branch point (BP) and polypyrimidine tract (PPT) we extracted 300nt upstream of the acceptor sites. The splice sites (SS) were scored with the Maximum Entropy approach (Yeo & Burge, 2004) and BPs and PPTs by a support vector machines method, SVM_BP (Corvelo et al, 2010), which gives independent and combined scores of BP and PPTs and is able to deal with distal BPs.

Immunoprecipitation of flag-tagged Matrin3

Flag-tagged Matrin3 wild-type and mPRI constructs were transfected into HEK-293T cells and 48h after transfection cells were harvested. The cell pellet was then lysed in 500uL of lysis buffer for 20min on ice. Cell extract was cleared by centrifugation at 12,000xg at 4C for 15min. 5% was kept as input and the remainder was added to 5ug of anti-flag antibody (SIGMA) pre-bound to protein G dynabeads (Invitrogen). Flag-tagged proteins were precipitated by rotating for 1h at 4C, followed by extensive washes and analysis by western blot. The following antibodies were used: p54nrb (BD biosciences), actin (Sigma), hnRNPF (Han et al, 2005), hnRNPL (Abcam), HuR (Santacruz), PSF (Santacruz) and PTB (Spellman et al, 2007).

References

Corvelo A, Hallegger M, Smith, CW, Eyras E (2010) Genome-wide association between branch point properties and alternative splicing. *PLoS Comput Biol* **6**, e1001016

Han K, Yeo G, An P, Burge CB, Grabowski PJ (2005) A combinatorial code for splicing silencing: UAGG and GGGG motifs. *Plos Biol* **3**: e158

Llorian M, Schwartz S, Clark TA, Hollander D, Tan LY, Spellman R, Gordon A, Schweitzer AC, de la Grange P, Ast G, Smith CW (2010) Position-dependent alternative splicing activity revealed by global profiling of alternative splicing events regulated by PTB. *Nat Struct Mol Biol* **17:** 1114-1123

Spellman R, Llorian M, Smith CW (2007) Crossregulation and functional redundancy between the splicing regulator PTB and its paralogs nPTB and ROD1. *Molecular cell* **27:** 420-434

Yeo G, Burge CB (2004) Maximum entropy modeling of short sequence motifs with applications to RNA splicing signals. *J Comput Biol* **11**, 377-94
