# Supplementary material for: Nuclear matrix protein Matrin3 regulates alternative splicing and forms overlapping regulatory networks with PTB
Source: EMBO J. 2015 Jan 19;34(5):653–68. doi: 10.15252/embj.201489852 (PMC4365034; doi:10.15252/embj.201489852)
Supplement: Supplementary file 5 [file embj0034-0653-sd5.pdf]

Supplementary Table S4. Over-represented 5mers in flanking introns of Matrin3 repressed exons.

The splicing event region, pentamer, total sites found, number of sequences with sites, log2(fold-ratio), p-value, FDR and logo are shown. Enriched pentamers were selected after multiple test correction (FDR < 0.05). See further details in main text Materials and Methods. Abbreviations, number of sequences comprised and over-represented RBP motifs for each splicing event region analyzed are shown at the foot of the table.

5mer enrichment analysis: Matrin3 NEG (-PTB)(Skipping)

region		5mer	sites	seq w/ sites	$\log_2$(ratio)	p-value	FDR	Logo
5IA	1	**TCTTG**	112	85 (44.7%)	1.22	3.65e-8	3.73e-5	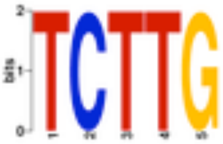
5IA	2	**TTTGT**	166	106 (55.8%)	1.06	7.29e-7	3.29e-4	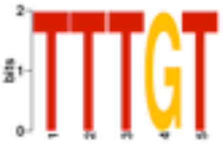
5IA	3	**TTCAT**	101	82 (43.2%)	1.07	1.24e-6	3.29e-4	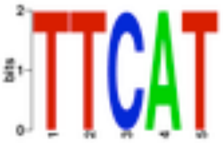
5IA	4	**TTTAT**	150	102 (53.7%)	1.03	1.28e-6	3.29e-4	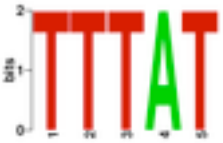
5IA	5	**TCATT**	103	80 (42.1%)	0.97	1.08e-5	2.20e-3	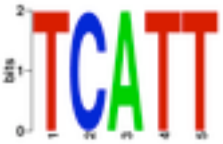
5IA	6	**TTCTT**	158	101 (53.2%)	0.93	1.37e-5	2.34e-3	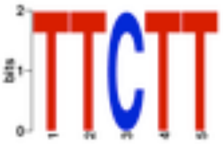

5IA	7	**CTTTG**	105	83 (43.7%)	0.92	2.43e-5	3.55e-3	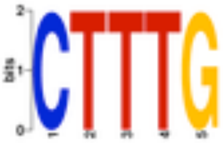
5IA	8	**TTTCT**	182	112 (58.9%)	0.89	3.17e-5	4.05e-3	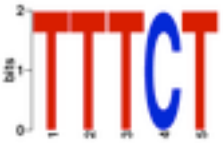
5IA	9	**CTCTA**	60	52 (27.4%)	1.03	4.64e-5	5.28e-3	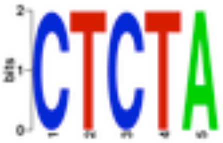
5IA	10	**CTCTT**	95	78 (41.1%)	0.89	5.21e-5	5.34e-3	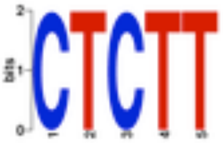
5IA	11	**TGTGA**	88	74 (38.9%)	0.88	1.08e-4	8.49e-3	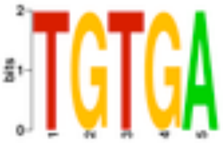
5IA	12	**TCTCT**	107	80 (42.1%)	0.84	1.37e-4	1.00e-2	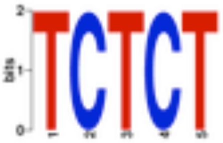
5IA	13	**GTTCT**	78	63 (33.2%)	0.9	1.47e-4	1.00e-2	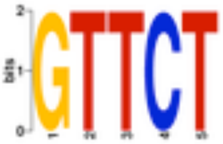
5IA	14	**CATTG**	68	58 (30.5%)	0.91	1.68e-4	1.00e-2	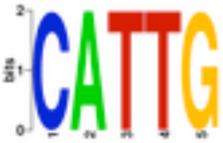
5IA	15	**TTCTG**	122	88 (46.3%)	0.81	1.76e-4	1.00e-2	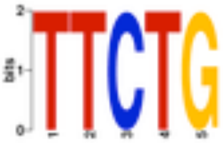
5IA	16	**TGTCT**	103	79 (41.6%)	0.83	1.76e-4	1.00e-2	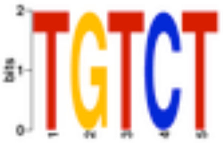

5IA	17	**TTGTC**	73	59 (31.1%)	0.89	2.10e-4	1.13e-2	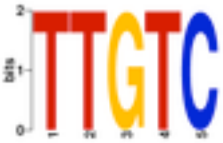
5IA	18	**CTGTA**	65	61 (32.1%)	0.84	3.06e-4	1.49e-2	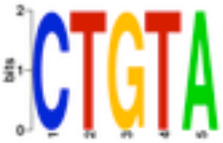
5IA	19	**TGACT**	64	57 (30%)	0.83	5.82e-4	2.31e-2	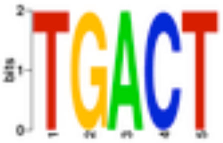
5IA	20	**ATAGT**	53	48 (25.3%)	0.87	5.88e-4	2.31e-2	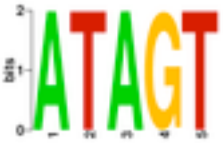
5IA	21	**TTATT**	127	89 (46.8%)	0.74	6.63e-4	2.52e-2	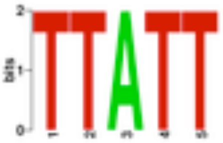
5IA	22	**ATACT**	51	47 (24.7%)	0.88	7.19e-4	2.60e-2	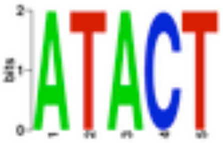
5IA	23	**CTTCT**	99	74 (38.9%)	0.75	7.36e-4	2.60e-2	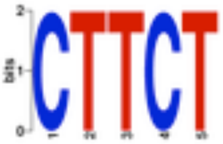
5IA	24	**TCTTT**	160	95 (50%)	0.71	8.76e-4	2.79e-2	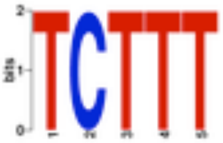
5IA	25	**TATCT**	66	54 (28.4%)	0.82	8.82e-4	2.79e-2	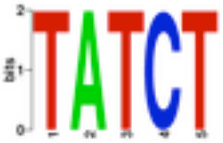
5IA	26	**ATTCT**	89	72 (37.9%)	0.74	8.96e-4	2.79e-2	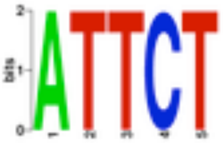

5IA	27	**TATTT**	153	99 (52.1%)	0.71	9.46e-4	2.79e-2	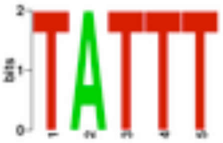
5IA	28	**TTATC**	55	48 (25.3%)	0.84	9.50e-4	2.79e-2	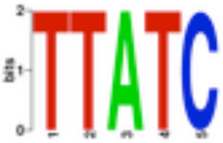
5IA	29	**GTTTA**	69	58 (30.5%)	0.8	9.73e-4	2.79e-2	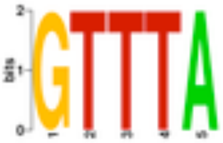
5IA	30	**TTGTA**	78	66 (34.7%)	0.75	1.08e-3	2.96e-2	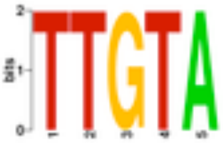
5IA	31	**CGTAC**	9	9 (4.7%)	1.93	1.10e-3	2.96e-2	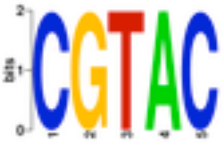
5IA	32	**CTGAT**	59	53 (27.9%)	0.81	1.16e-3	3.04e-2	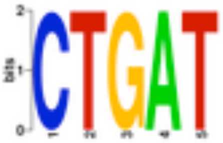
5IA	33	**TTATA**	90	67 (35.3%)	0.73	1.22e-3	3.09e-2	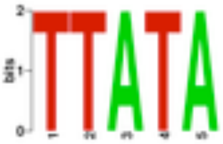
5IA	34	**ATGTG**	81	64 (33.7%)	0.75	1.27e-3	3.09e-2	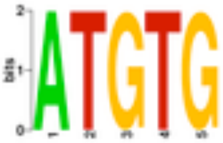
5IA	35	**ACTTC**	53	48 (25.3%)	0.83	1.31e-3	3.13e-2	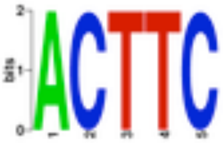
5IA	36	**GTATT**	72	58 (30.5%)	0.76	1.47e-3	3.42e-2	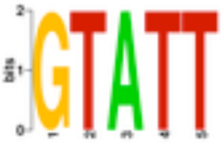

5IA	37	**GATGA**	51	49 (25.8%)	0.81	1.56e-3	3.54e-2	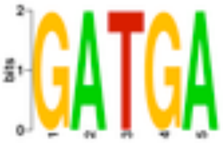
5IA	38	**TACTG**	49	47 (24.7%)	0.79	1.98e-3	4.31e-2	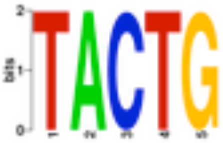
5IA	39	**GTCTT**	82	61 (32.1%)	0.72	2.07e-3	4.43e-2	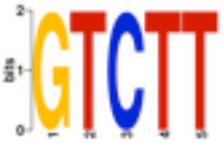
5IA	40	**TCCTT**	95	72 (37.9%)	0.68	2.35e-3	4.91e-2	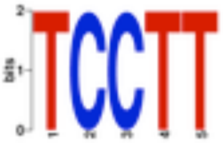
3ID	1	**TTCTT**	171	114 (59.1%)	1.09	2.48e-7	2.54e-4	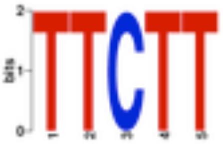
3ID	2	**TCCTT**	125	90 (46.6%)	1.06	9.22e-7	4.72e-4	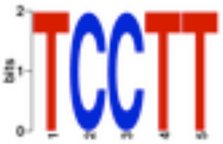
3ID	3	**CCTTA**	70	58 (30.1%)	1.17	1.52e-6	5.19e-4	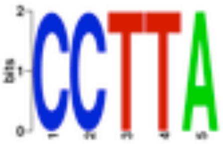
3ID	4	**TCTTT**	168	110 (57%)	1	2.47e-6	6.31e-4	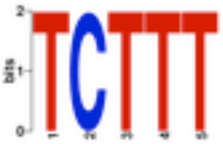
3ID	5	**TTTTC**	201	115 (59.6%)	0.99	3.11e-6	6.31e-4	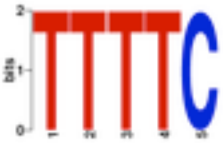

3ID	6	**TTTCT**	230	124 (64.2%)	1	3.70e-6	6.31e-4	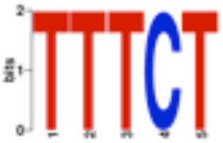
3ID	7	**CTTAC**	56	51 (26.4%)	1.14	8.87e-6	1.30e-3	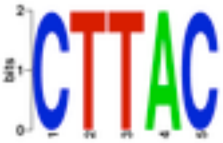
3ID	8	**CATCT**	97	76 (39.4%)	0.97	1.61e-5	2.06e-3	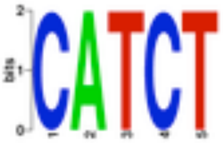
3ID	9	**TCTCT**	132	90 (46.6%)	0.87	4.99e-5	5.29e-3	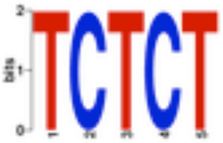
3ID	10	**CTTTT**	183	113 (58.5%)	0.86	5.17e-5	5.29e-3	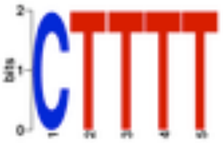
3ID	11	**TCTGT**	119	95 (49.2%)	0.85	6.85e-5	5.85e-3	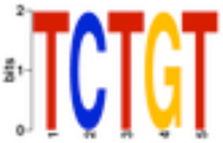
3ID	12	**TGTCT**	102	82 (42.5%)	0.85	1.12e-4	8.85e-3	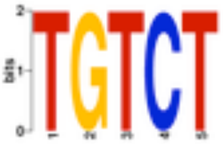
3ID	13	**TTCAT**	115	81 (42%)	0.82	1.58e-4	1.15e-2	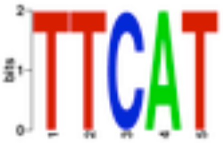
3ID	14	**TTCTC**	123	88 (45.6%)	0.8	1.85e-4	1.26e-2	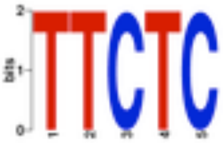
3ID	15	**ATCTT**	99	72 (37.3%)	0.83	2.08e-4	1.33e-2	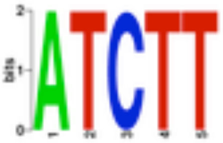

3ID	16	**TCTTC**	108	74 (38.3%)	0.8	2.74e-4	1.57e-2	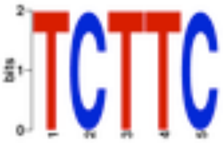
3ID	17	**TATCT**	71	61 (31.6%)	0.87	2.75e-4	1.57e-2	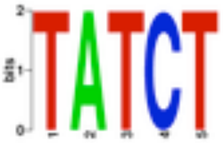
3ID	18	**ATCCT**	67	59 (30.6%)	0.87	3.18e-4	1.72e-2	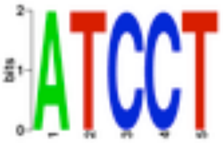
3ID	19	**ATCTG**	78	66 (34.2%)	0.81	3.84e-4	1.96e-2	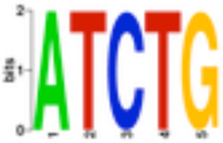
3ID	20	**CTCTT**	100	80 (41.5%)	0.77	4.29e-4	2.09e-2	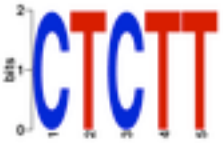
3ID	21	**TTTCC**	113	87 (45.1%)	0.74	4.85e-4	2.26e-2	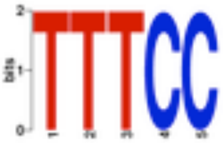
3ID	22	**CATTC**	68	60 (31.1%)	0.81	5.81e-4	2.31e-2	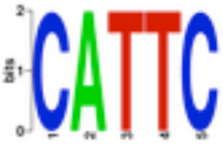
3ID	23	**TTCTG**	126	92 (47.7%)	0.73	5.96e-4	2.31e-2	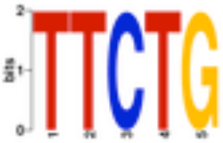
3ID	24	**TACTT**	84	67 (34.7%)	0.78	6.09e-4	2.31e-2	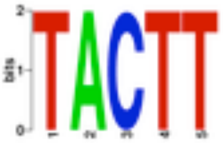
3ID	25	**TTACT**	73	67 (34.7%)	0.78	6.09e-4	2.31e-2	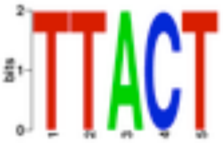

3ID	26	**GTTTT**	146	97 (50.3%)	0.71	7.32e-4	2.68e-2	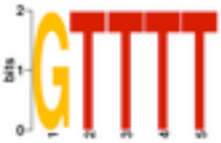
3ID	27	**CTATC**	36	34 (17.6%)	0.99	8.46e-4	2.99e-2	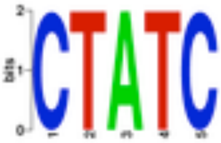
3ID	28	**GTACA**	42	41 (21.2%)	0.91	9.52e-4	3.25e-2	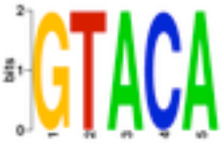
3ID	29	**TTGGT**	85	64 (33.2%)	0.74	1.35e-3	4.45e-2	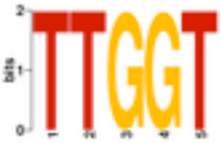
3ID	30	**TTTAC**	73	61 (31.6%)	0.75	1.43e-3	4.58e-2	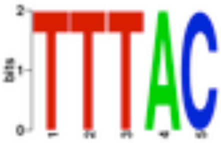
3IA	1	**TCTTA**	86	71 (36.8%)	1.02	7.20e-6	3.69e-3	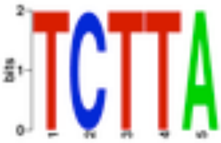
3IA	2	**TTATT**	128	94 (48.7%)	0.88	4.30e-5	9.18e-3	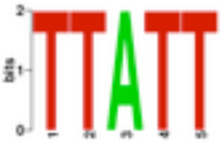
3IA	3	**GATAG**	47	41 (21.2%)	1.12	4.63e-5	9.18e-3	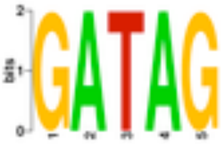
3IA	4	**GTTTT**	127	95 (49.2%)	0.86	4.77e-5	9.18e-3	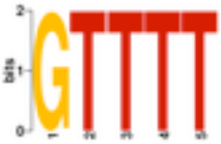

3IA	5	**ATTCA**	86	65 (33.7%)	0.94	5.38e-5	9.18e-3	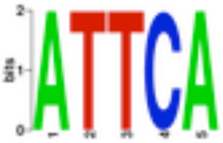
3IA	6	**ATTAA**	108	74 (38.3%)	0.89	7.38e-5	1.08e-2	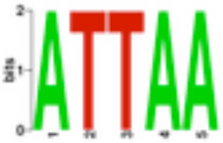
3IA	7	**AATTC**	63	58 (30.1%)	0.92	1.17e-4	1.50e-2	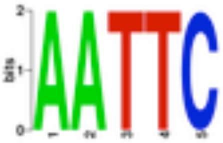
3IA	8	**ATATT**	122	85 (44%)	0.81	1.55e-4	1.60e-2	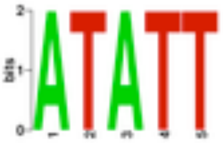
3IA	9	**TATTT**	163	106 (54.9%)	0.8	1.57e-4	1.60e-2	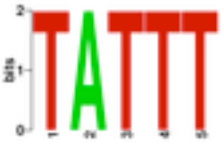
3IA	10	**ATGAC**	51	46 (23.8%)	0.98	1.98e-4	1.60e-2	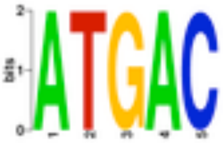
3IA	11	**TTCTT**	137	96 (49.7%)	0.79	2.03e-4	1.60e-2	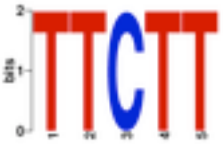
3IA	12	**TCAAT**	48	44 (22.8%)	0.96	3.59e-4	2.35e-2	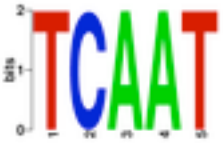
3IA	13	**TATGT**	84	67 (34.7%)	0.82	3.86e-4	2.35e-2	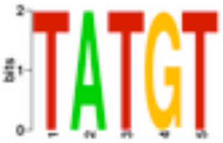
3IA	14	**CATTC**	64	54 (28%)	0.87	3.88e-4	2.35e-2	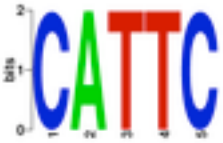

3IA	15	**TTTTT**	186	106 (54.9%)	0.75	3.90e-4	2.35e-2	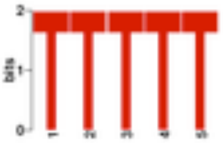
3IA	16	**TAAAT**	112	83 (43%)	0.76	5.04e-4	2.87e-2	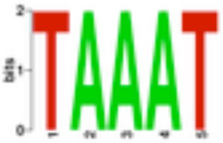
3IA	17	**CGTTG**	17	16 (8.3%)	1.44	6.85e-4	3.44e-2	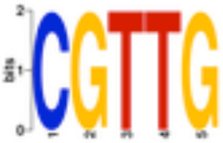
3IA	18	**GATAA**	58	48 (24.9%)	0.85	8.84e-4	4.00e-2	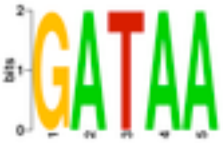
3IA	19	**AAGTT**	78	64 (33.2%)	0.77	8.98e-4	4.00e-2	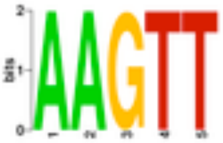
3IA	20	**AAATG**	121	89 (46.1%)	0.7	9.77e-4	4.17e-2	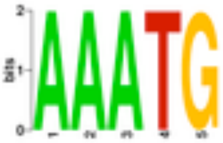
3IA	21	**TTTAA**	152	97 (50.3%)	0.69	1.27e-3	4.99e-2	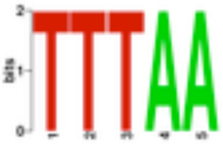
3IA	22	**CGGTA**	10	10 (5.2%)	1.78	1.28e-3	4.99e-2	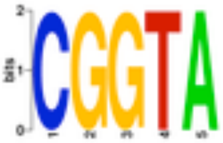
3IA	23	**TATCT**	61	54 (28%)	0.78	1.39e-3	4.99e-2	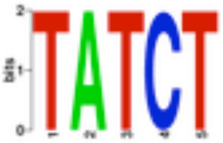
3IA	24	**ACTTT**	90	74 (38.3%)	0.71	1.46e-3	4.99e-2	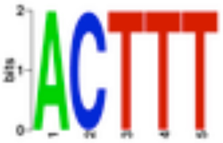

3IA	25	**TAAAA**	138	93 (48.2%)	0.68	1.48e-3	4.99e-2	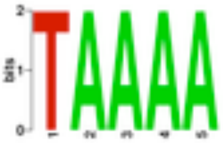

5' Exon (5E), # sequences = 198, # 5mers = 0
5' Intron Donor (5ID), # sequences = 190, # 5mers = 0
5' Intron Acceptor (5IA), # sequences = 190, # 5mers = 40
Cassette Exon (CE), # sequences = 189, # 5mers = 0
3' Intron Donor (3ID), # sequences = 193, # 5mers = 30
3' Intron Acceptor (3IA), # sequences = 193, # 5mers = 25
3' Exon (3E), # sequences = 198, # 5mers = 0
