# Supplementary material for: Nuclear matrix protein Matrin3 regulates alternative splicing and forms overlapping regulatory networks with PTB
Source: EMBO J. 2015 Jan 19;34(5):653–68. doi: 10.15252/embj.201489852 (PMC4365034; doi:10.15252/embj.201489852)
Supplement: Supplementary file 6 [file embj0034-0653-sd6.pdf]

Supplementary Table S5. Over-represented RBP binding motifs obtained by RNA-compete (Ray *et al*. 2013) in flanking introns of Matrin3 repressed exons.

The splicing event region, RBP gene name, RNA-compete id, species of the RBP, total sites found, number of sequences with sites, log2(fold-ratio), p-value, FDR and sites logo are shown. Enriched motifs were selected after multiple test correction (FDR < 0.05). Motifs shown here include those for all RBPs (multiple species) from Ray *et al.* 2013, whereas Figure 4 shows only the enriched motifs for human proteins. See further details in main text Materials and Methods. Abbreviations, number of sequences comprised and over-represented RBP motifs for each splicing event region analyzed are shown at the foot of the table.

RBP motifs enrichment analysis: Matrin3 NEG (-PTB)(Skipping)

region		gene name	RBP id	species	sites	seq w/ sites	$\log_2$(ratio)	p-value	FDR	Logo
5IA	1	MATR3	**MATR3_00037**	*Homo sapiens*	83	67 (35.3%)	1.1	1.85e-6	4.52e-4	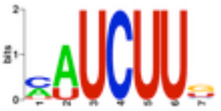
5IA	2	Tv_0258	**Tv_0258_00258**	*Trichomonas vaginalis*	124	86 (45.3%)	0.77	4.15e-4	3.38e-2	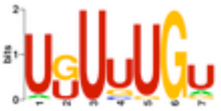
3ID	1	PTBP1	**PTBP1_00269**	*Homo sapiens*	222	124 (64.2%)	1.24	6.80e-9	1.66e-6	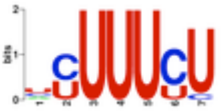
3ID	2	PCBP1	**PCBP1_00239**	*Mus musculus*	143	96 (49.7%)	0.94	1.05e-5	7.32e-4	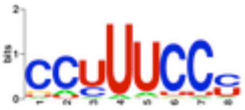
3ID	3	PTBP1	**PTBP1_00268**	*Homo sapiens*	255	120 (62.2%)	0.94	1.08e-5	7.32e-4	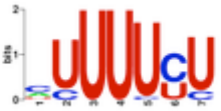
3ID	4	Tb_0220	**Tb_0220_00220**	*Trypanosoma brucei*	181	112 (58%)	0.93	1.20e-5	7.32e-4	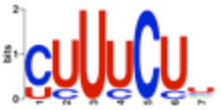
3ID	5	FNE	**FNE_00120**	*Drosophila melanogaster*	102	76 (39.4%)	0.86	1.02e-4	3.55e-3	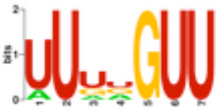
3ID	6	CG5213	**CG5213_00010**	*Drosophila melanogaster*	128	93 (48.2%)	0.78	2.49e-4	7.59e-3	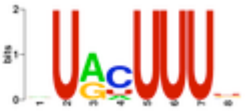
3ID	7	U2AF50	**U2AF50_00080**	*Drosophila melanogaster*	377	74 (38.3%)	0.8	3.65e-4	9.91e-3	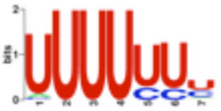
3ID	8	MATR3	**MATR3_00037**	*Homo sapiens*	89	64 (33.2%)	0.83	4.09e-4	9.98e-3	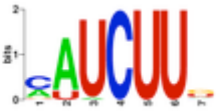

3IA	1	HuR	**HuR_00032**	*Homo sapiens*	212	103 (53.4%)	0.88	3.50e-5	8.55e-3	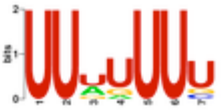
3IA	2	Hrp1p	**Hrp1p_00031**	*Saccharomyces cerevisiae*	60	49 (25.4%)	0.99	1.27e-4	1.55e-2	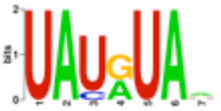
3IA	3	RBP9	**RBP9_00132**	*Drosophila melanogaster*	99	76 (39.4%)	0.8	3.01e-4	2.45e-2	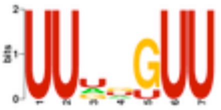
3IA	4	HuR	**HuR_00136**	*Homo sapiens*	140	90 (46.6%)	0.72	7.62e-4	4.08e-2	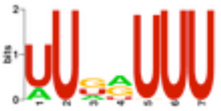
3IA	5	ZCRB1	**ZCRB1_00087**	*Homo sapiens*	65	50 (25.9%)	0.83	8.37e-4	4.08e-2	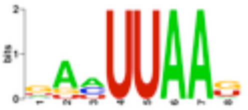
3IA	6	SXL	**SXL_00119**	*Drosophila melanogaster*	220	73 (37.8%)	0.72	1.34e-3	4.57e-2	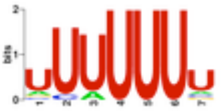
3IA	7	PFI1435w	**PFI1435w_00202**	*Plasmodium falciparum*	217	92 (47.7%)	0.67	1.48e-3	4.57e-2	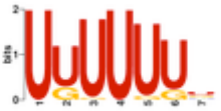
3IA	8	FNE	**FNE_00120**	*Drosophila melanogaster*	79	65 (33.7%)	0.72	1.50e-3	4.57e-2	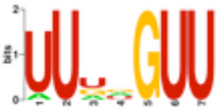
3IA	9	EXC-7	**EXC-7_00014**	*Caenorhabditis elegans*	67	60 (31.1%)	0.73	1.84e-3	5.00e-2	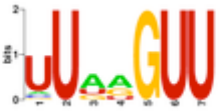

5' Exon (5E), # sequences = 198, # RBPs = 0
5' Intron Donor (5ID), # sequences = 190, # RBPs = 0
5' Intron Acceptor (5IA), # sequences = 190, # RBPs = 2
Cassette Exon (CE), # sequences = 189, # RBPs = 0
3' Intron Donor (3ID), # sequences = 193, # RBPs = 8
3' Intron Acceptor (3IA), # sequences = 193, # RBPs = 9
3' Exon (3E), # sequences = 198, # RBPs = 0
