# Supplementary material for: Nuclear matrix protein Matrin3 regulates alternative splicing and forms overlapping regulatory networks with PTB
Source: EMBO J. 2015 Jan 19;34(5):653–68. doi: 10.15252/embj.201489852 (PMC4365034; doi:10.15252/embj.201489852)
Supplement: Supplementary file 7 [file embj0034-0653-sd7.pdf]

THE EMBO JOURNAL

Manuscript EMBO-2014-89852

Nuclear Matrix Protein Matrin3 regulates alternative splicing and forms overlapping regulators networks with PTB

Miguel B Coelho, Jan Attig, Nicolas Bellora, Julian König, Martina Hallegger, Melis Kayikci, Eduardo Eyras, Jernej Ule and Christopher W.J. Smith

Corresponding author: Christopher W.J. Smith, University of Cambridge

Review timeline:

Submission date:	19 August 2014
Editorial Decision:	18 October 2014
Revision received:	28 November 2014
Editorial Decision:	10 December 2014
Revision received:	12 December 2014
Accepted:	15 December 2014

Editor: Anne Nielsen

Transaction Report:

(Note: With the exception of the correction of typographical or spelling errors that could be a source of ambiguity, letters and reports are not edited. The original formatting of letters and referee reports may not be reflected in this compilation.)

1st Editorial Decision 18 October 2014

Thank you for submitting your manuscript for consideration by the EMBO Journal. It has now been seen by three referees whose comments are shown below.

As you will see from the reports, all referees express interest in the findings reported in your manuscript. However, they also do raise a number of specific criticisms that you will have to address before they can support publication of the manuscript. While some of them relate mainly to data presentation, you will also need to include further experimental data to conclusively support the role for Matrin3 in splicing.

For the revised manuscript I would particularly ask you to focus your efforts on the following issues:

-> the technical point raised by ref#1 concerning the conclusions derived from the Matrin3 iCLIP binding pattern (ref#1 points 1 and 2)

-> the functional interplay between Matrin3, PTB and other putative PTB interactors (ref #1 points 3 and 4, ref#2)

-> In addition, while it would clearly strengthen the study to include data from other cell types (as pointed out by ref #2), this will not be an absolute requirement from our side. However, we do encourage you to include any data you may have at hand to extend the study beyond HeLa cells.

Given the referees' positive recommendations, I would like to invite you to submit a revised version of the manuscript, addressing the comments of all three reviewers. I should add that it is EMBO Journal policy to allow only a single round of revision, and acceptance of your manuscript will therefore depend on the completeness of your responses in this revised version.

When preparing your letter of response to the referees' comments, please bear in mind that this will form part of the Review Process File, and will therefore be available online to the community. For more details on our Transparent Editorial Process, please visit our website: http://emboj.embopress.org/about#Transparent_Process

We generally allow three months as standard revision time. As a matter of policy, competing manuscripts published during this period will not negatively impact on our assessment of the conceptual advance presented by your study. However, we request that you contact the editor as soon as possible upon publication of any related work, to discuss how to proceed. Should you foresee a problem in meeting this three-month deadline, please let us know in advance and we may be able to grant an extension.

Thank you for the opportunity to consider your work for publication. I look forward to your revision.

REFEREE REPORTS

Referee #1:

This study from the Smith lab examines the function of the Matrin3 protein, an abundant RNA binding protein found associated with the nuclear matrix that has been implicated in a wide variety of nuclear functions. The authors identified Matrin3 as interacting the splicing regulator PTBP1 in GST pull down assays. They map this interaction to a specific peptide (PRI motif) in Matrin3 with strong similarity to peptides known to mediate the interaction of Raver1 protein to PTB. Using exon junction microarrays, they identify a set of splicing changes occurring across the transcriptome after knock down of Matrin3 in Hela cells. This is compared to splicing events regulated by PTB, but only minimal overlap is found between these two target sets and they do not seem to find any exons that might be synergistically affected by the two proteins. Analyzing the exons regulated by Matrin3, they find that their adjacent introns are slightly longer on average than for PTB regulated exons. These introns are enriched in a previously characterized binding motif for Matrin3 as well as for several other pyrimidine rich motifs. Performing CLIPseq to identify genomewide Matrin3 and PTB binding sites, they examine binding of these two proteins adjacent to exons regulated by them. They find an unusual pattern of binding for Matrin3 adjacent to its putative target exons. Rather than clear peaks of localized binding that would indicate a preferred binding site, the authors observe a broad distribution of the isolated CLIP tags that are evenly distributed across the region containing the alternative exon. Examining two Matrin3 target exons in reporter gene assays they analyze the structures needed in coexpressed Matrin3 to alter splicing. They show that both the PRI motif and the RRMs are needed for Matrin3 activity. Interestingly, loss of one of the Zn finger domains seems to result in enhanced activity.

There is a long literature on Matrin3 with a variety of disparate findings. This study presents new data that are of value in thinking about Matrin3 function and points out studies that might need to be reassessed from these data. I think the paper will be a useful contribution to the literature. However the authors make several interpretations of the data that are not necessarily supported by it. These should be corrected, along with other editorial changes listed below.

1. On page 10 and later in the discussion, the authors interpret the extended distribution of tags seen in the CLIP as indicating extended regions of non-specific binding by the protein and invoke a model that the initial binding of Matrin3 to a specific site nucleates the binding of multiple matrin3 proteins across an extended region. This model has been proposed for other proteins but is not yet well demonstrated. In fact, the proteins mentioned as having this extended binding do not show the CLIP pattern seen here. In the data presented here, the pattern of CLIP tags could arise from random non-sequence specific binding of single proteins across the region. Such a pattern could occur if the

Matrin3 was in close proximity to the region, perhaps bound to chromatin or nuclear matrix, and was trapped by crosslinking in a variety of non-specific interactions along the RNA.

2. The statement that Matrin3 is clearly a direct splicing repressor is also not well supported. The authors show that repressed exons have increased crosslinking, but since no specific site can be mutated, they cannot test if this binding is needed for the repression seen. Alternatively, while they observe enriched binding around exons, they do not discuss how well this enriched binding correlates with effects on splicing. Are there exons with Matrin3 binding that are not affected in the knockdown? And exons affected by the knockdown that do not have matrin3 enrichment?

3. In the description of Figure 6 on page 11, the ABI2 data is in 6D not 6C and the activity was increased by deletion of ZF1 not ZF2. The fact that the PRI mutation affects the activity does not necessarily implicate PTB as suggested, as many other proteins might also bind this peptide. A more informative experiment might be to test co-expression of PTB with wt and mPRI matrin3 to assess whether an effect of PTB might be lost in the mPRI mutant. Although this experiment could be difficult in the presence of background expression of both proteins. Alternatively, one could measure the percent of matrin3 that was bound by PTB in vivo.

4. Note also in this Figure and elsewhere, the authors present splicing analyses using capillary electrophoresis output. This is a valid assay but the data are often difficult to read as they look like drawings from an illustration program. For Figure 6C in particular, the changes in intensity based on the drawing gray scale are almost impossible to see. The data would be more convincing if they could find another way to present this.

5. The authors discuss their newly identified PTB interacting proteins in relation to others previously identified. Another protein that is discussed as a PTB interactor but not described in the pulldown is MRG15. Was this also negative in their assay?

6. The supplemental data tables are not described as far as I could tell. What is the table showing motifs from different species? Each of these tables should have a descriptive paragraph.

Referee #2:

The authors identified matrin3 as an interacter of PTB and go on to demonstrate that matrin3 is a regulator of alternative splicing. Matrin3 is an abundant inner nuclear matrix protein and has been implicated in a number of nuclear functions. It interacts with several RNA binding proteins that regulate splicing but has not been demonstrated to be a splicing regulator.

A GST fusion protein of PTB RRM2 was used to pull out interactors from HeLa nuclear extracts (nicely comparing wt to a single residue mutant as control). Multiple interacting proteins were identified and this manuscript focuses on matrin3. The major result are that knock down of matrin3 in HeLa cells results in altered splicing of >600 splicing events, the majority of which are repressed by matrin3. Second, while there is significant enrichment for events that are co-regulated by PTB and matrin3, the majority are regulated by matrin3 and not PTB. The finding that alternative splicing of a large number of genes is affected by loss of matrin3 is significant. The next step is to determine what fraction are direct and then to determine the relevance of matrin3 regulated splicing events. The results from analysis of exon features and enriched motifs showed little of significance for the smaller number of events activated by loss of matrin3. The authors conclude that reduced splicing in response to loss of matrin3 could be indirect. However, exons repressed by matrin3 tended to be flanked by longer introns than expected by chance. In addition, pyrimidine rich motifs were enriched near repressed exons.

iCLIP analysis revealed an unusual pattern in which matrin3 binding was located along long regions of the introns and extending into the repressed exon. It is interesting that this is in contrast to the standard pattern of RNA binding proteins producing peaks of binding downstream of exons that are activated and upstream of exons that are repressed by binding. There is clearly something to be learned from this pattern. The pattern of PTB binding by iCLIP showed binding of PTB to matrin3-regulated, but PTB-independent exons suggested that matrin3 recruits PTB to the intron. Since these exons are defined as PTB-independent, the significance of this observation is unclear.

The authors used minigene experiments and matrin3 deletions to demonstrate that the RRM is required for splicing activity. This is relevant since the difference in iCLIP results from other RNA binding proteins could reflect differences in the domain required for RNA binding. A particularly strong result is that mutation of the PRI motif, which interacts with PTB as well as other proteins, ablates matrin3 activity even on a PTB-independent exon. This result suggests that protein-protein interactions with endogenous proteins is impotent for the exogenous matrin3 function.

The authors have identified matrin3 as a regulator of alternative splicing. There is also a non-standard mechanisms of binding within cells based on iCLIP and therefore it is likely that there is important new information to be gleaned. Similarly the requirement for the PRI motif is very interesting.

There are several novel aspects to this manuscript; here are my concerns;
1. The results are limited to HeLa cells. While this establishes a role for matrin3 in splicing in a general sense, this does not address any potential for broader significance in normal or abnormal cell function.
There is clearly something of interest in the binding of matrin3 to it's target RNAs based on iCLIP. This appears to be related to the fact that mutation of the PRI is required for activity. Identification of the PRI-interacting proteins that are relevant to matrin3 activity would add significantly to the manuscript.

Referee #3:

The manuscript by Coelho et al describes an interesting study that shows the role of Matrin 3 in modulating alternative splicing in a large group of genes.
The take home message is clear and mostly supported by the data. However there are serious inconsistencies (outlined below) between text and data. Obviously something has gone wrong on the compilation of the manuscript.

1- There are serious inconsistencies between the data described in Fig 3, the Figure 6 and its description in page 11, that cause considerable confusion. The text in page 11 describes the experiments carried out by co transfection of mingene reporters and Matrin 3 variants . While the text refers to AB12 exon 9 and ST 7 exon 11 figure 6 shows ST7 exon 7 and AB12 exon 8 while figure 3 talks about ST7 exon 11. Ignoring this and interpreting the figure directly I think have understood the results. However the whole section on Page 11 is not consistent with the figure and should be re written and corrected.

2- In the same page, final sentence, the authors state that Matrin 3 requires both its RRMs to act as splicing regulators but they have not tested single deletions, the lack of only one of the RRMs may conserve full function. The statement should be modified accordingly.

3- In the Discussion the authors could make some considerations on the possible different activities of the RRMs. Likewise although some of the zinc finger domains have considerable impact on the activity (Fig 6) they are mostly ignored through the results and discussion.

Minor typo: Legend to Figure 3A ".....Matrin 3, PTB and nPTB (lane 7) the 7 is missing"

1st Revision - authors' response 28 November 2014

EMBOJ-2014-89852
Responses to referees
We thank all three referees for their positive reviews and for their constructive criticisms of the manuscript.

REFEREE 1

1. "*On page 10 and later in the discussion, the authors interpret the extended distribution of tags seen in the CLIP as indicating extended regions of non-specific binding by the protein and invoke a model that the initial binding of Matrin3 to a specific site nucleates the binding of multiple matrin3 proteins across an extended region. This model has been proposed for other proteins but is not yet well demonstrated. In fact, the proteins mentioned as having this extended binding do not show the CLIP pattern seen here. In the data presented here, the pattern of CLIP tags could arise from random non-sequence specific binding of single proteins across the region. Such a pattern could occur if the Matrin3 was in close proximity to the region, perhaps bound to chromatin or nuclear matrix, and was trapped by crosslinking in a variety of non-specific interactions along the RNA.*"

Response: The referee is correct to point out that the RNA maps for hnRNP A1 and PTB show distinct peaks. However, numerous analyses of model events have indicated that this is not a general mechanism for these proteins. In contrast, our splicing map for Matrin3 is consistent with such a model, and suggests that for Matrin3, this could be its general mode of action. The dependence on its RNA but not DNA binding domains, combined with the observed enrichment of sequence motifs corresponding to optimal sites for the RRM domains, make this a more likely explanation than completely non-specific RNA contact mediated by chromatin binding. Nevertheless, we accept that this model will require future experimental testing using a variety of methods including *in vitro* experiments with recombinant Matrin3. Although we can prepare recombinant Matrin3 RRMs (as used in the Ray et al RNA-compete paper), we are having problems expressing larger fragments of the protein that retain activity *in vivo*. So we are not yet in a position to test the model rigorously.

However, we have modified the discussion text as follows:

Similar models were originally suggested for repression by PTB (Wagner & Garcia-Blanco, 2001) and hnRNPA1 (Zhu et al, 2001), but analysis of numerous model systems has shown that this is not a common mechanism for these proteins (e.g. (Cherny et al, 2010)), and their splicing maps show distinct peaks of enriched binding (Huelga et al, 2012; Llorian et al, 2010; Xue et al, 2009). In contrast, the Matrin3 splicing map (Fig 5A) is consistent with a general mechanism of action in which initial binding of Matrin3 at specific sites is followed by propagative binding across a wide region of RNA, leading to repression of the targeted exon. This model is amenable to testing by various methods including single molecule analysis (Cherny et al, 2010).

2. "*The statement that Matrin3 is clearly a direct splicing repressor is also not well supported. The authors show that repressed exons have increased crosslinking, but since no specific site can be mutated, they cannot test if this binding is needed for the repression seen. Alternatively, while they observe enriched binding around exons, they do not discuss how well this enriched binding correlates with effects on splicing. Are there exons with Matrin3 binding that are not affected in the knockdown? And exons affected by the knockdown that do not have matrin3 enrichment?*"

Response: We have modified the text to moderate the statements about the evidence for direct action as a splicing repressor, including removing the word "direct" from the abstract. We tested for a correlation between the density of Matrin3 CLIP tags and predicted magnitude of splicing changes upon knockdown, but found no clear correlation. This is probably explained by non-saturation of the CLIP library. We have now included details of a statistical test that indicates that the high degree of enrichment of Matrin3 binding around repressed exons (blue trace in Fig 5A) compared to unregulated exons (grey trace in Fig 5A) is statistically signficant. This is now noted in the associated Results text:

We did not observe a clear correlation between tag density and degree of splicing change upon Matrin3 knockdown, which may be related to lack of saturation of the iCLIP library. Nevertheless, the density of Matrin3 tags around repressed compared to control exons was highly significant ($p < 0.0001$, χ^2 test).

We have also modified the Discussion to indicate that further experimental data will be required to substantiate the models for direct repression:

Matrin3 function as a direct splicing repressor is supported by its observed binding around repressed exons (Fig. 5) along with the enrichment in flanking intron segments of optimal binding motifs for its RRM motifs (Fig. 4B). Consistent with this, it requires intact RNA binding domains, but not DNA binding domains, for its splicing repressor activity (Fig. 6). Nevertheless, further evidence of its direct

mode of action could be provided by *in vitro* analyses of its binding to regulated RNAs, demonstration that specific binding sites are required for its splicing repressor activity, and more detailed analysis of the roles of the individual RRM domains.

3. *In the description of Figure 6 on page 11, the ABI2 data is in 6D not 6C and the activity was increased by deletion of ZF1 not ZF2. The fact that the PRI mutation affects the activity does not necessarily implicate PTB as suggested, as many other proteins might also bind this peptide. A more informative experiment might be to test co-expression of PTB with wt and mPRI matrin3 to assess whether an effect of PTB might be lost in the mPRI mutant. Although this experiment could be difficult in the presence of background expression of both proteins. Alternatively, one could measure the percent of matrin3 that was bound by PTB in vivo.*

Response: Minor corrections have been made. As ABI2 exon 8 is regulated by both PTB and Matrin3 the effect of the PRI mutant is most readily explained by the disruption of PTB binding. This is the simplest explanation that is consistent with all the available data (we currently have not identified other PRI-interacting proteins). Nevertheless, we take the referees point and have included additional new experimental data (Fig 6 E-G). The new data shows that PTB knockdown has no effect upon the Matrin3 repression of the PTB-independent ST7 exon 11. In contrast, Matrin3 has no effect on ABI2 exon 8 upon PTB depletion, suggesting that its main role is to antagonize PTB activity at this exon.
We attempted further experiments as suggested by the referee, combining knockdown of Matrin3, over-expression of PTB and over-expression of WT or PRI mutant Matrin3. However, as anticipated by the referee, it was difficult to obtain meaningful data by over-expression of two proteins in cells where both are already expressed. We suspect that to address this properly in the future we will need to use inducible FLIP-in cell lines.
We did not pursue the suggestion of measuring the precentage of Matrin3 bound to PTB. Given its known enrichment in nuclear matrix fractions we are confident that a large proportion of Matrin3 would not be associated with PTB. It therefore seemed unlikely that this experiment would provide useful insights in this instance.

4. *Note also in this Figure and elsewhere, the authors present splicing analyses using capillary electrophoresis output. This is a valid assay but the data are often difficult to read as they look like drawings from an illustration program. For Figure 6C in particular, the changes in intensity based on the drawing gray scale are almost impossible to see. The data would be more convincing if they could find another way to present this.*

Response: we have replaced the QIAxcel gel images in Fig 6 with histograms.

5. *The authors discuss their newly identified PTB interacting proteins in relation to others previously identified. Another protein that is discussed as a PTB interactor but not described in the pulldown is MRG15. Was this also negative in their assay?*

Response: MRG15 was not detected in our pulldowns. We agree that because of its reported functional association with PTB, this is worth noting. This is now stated explicitly in the Discussion (p15).

6. *The supplemental data tables are not described as far as I could tell. What is the table showing motifs from different species? Each of these tables should have a descriptive paragraph.*

Response: Appropriate descriptions have been added to the Tables. We apologize for this oversight.

REFEREE 2

1. *The results are limited to HeLa cells. While this establishes a role for matrin3 in splicing in a general sense, this does not address any potential for broader significance in normal or abnormal cell function.*
Response: we agree that in the future it will be interesting to pursue the function of Matrin3 in cell types that are more relevant for its normal physiological function and for its altered function in disease.

2. *Identification of the PRI-interacting proteins that are relevant to matrin3 activity would add significantly to the manuscript.*

Response: We agree that it will be of great interest to identify proteins other than PTB that interact with the Matrin3 PRI. We have carried out some co-IP mass spectrometry experiments comparing wild-type and PRI mutant Matrin3, but the only clear differences we have observed were in PTB binding. We also analyzed co-IP by western blot for candidate Matrin3-interactors including PSF, HuR, p54nrb and hnRNPF. We saw no other proteins that responded in the same way as PTB, although we did observe that some proteins showed increased association with Matrin3 PRI mutant. This suggests that some of these other proteins interact with Matrin3 in a mutually exclusive way with PTB, albeit not using the PRI. We have included the co-IP western as Extended View Figure 5 and referred to this data in the Discussion.

REFEREE 3

1.*There are serious inconsistencies between the data described in Fig 3, the Figure 6 and its description in page 11, that cause considerable confusion. The text in page 11 describes the experiments carried out by co transfection of mingene reporters and Matrin 3 variants . While the text refers to AB12 exon 9 and ST 7 exon 11 figure 6 shows ST7 exon 7 and AB12 exon 8 while figure 3 talks about ST7 exon 11. Ignoring this and interpreting the figure directly I think have understood the results. However the whole section on Page 11 is not consistent with the figure and should be re written and corrected.*

Response: we apologize for the confusion with exon numbers. We have made the appropriate corrections throughout.

2.*In the same page, final sentence, the authors state that Matrin 3 requires both its RRMs to act as splicing regulators but they have not tested single deletions, the lack of only one of the RRMs may conserve full function. The statement should be modified accordingly.*

Response: the correction has been made.

3. *In the Discussion the authors could make some considerations on the possible different activities of the RRMs. Likewise although some of the zinc finger domains have considerable impact on the activity (Fig 6) they are mostly ignored through the results and discussion.*

Response: we think that it would be premature to discuss possible differences in the activities of the RRMs without first carrying out the experiments. In a modified section of the Discussion we have noted the need for more detailed analysis of the individual RRMs, and also briefly discussed the unexpected effect of deleting the ZF1 DNA binding domain.

Nevertheless, further evidence of its direct mode of action could be provided by in vitro analyses of its binding to regulated RNAs, demonstration that specific binding sites are required for its splicing repressor activity, and more detailed analysis of the roles of the individual RRM domains. Unexpectedly, deletion of the ZF1 DNA binding domain actually enhanced the activity of transfected Matrin3 (Fig. 6). One possible explanation for this observation is that deletion of ZF1 affects the distribution of Matrin3 between pools that are active for splicing regulation or that are localized elsewhere.

Minor typo: Legend to Figure 3A ".....Matrin 3, PTB and nPTB (lane 7) the 7 is missing"

Response: the correction has been made

2nd Editorial Decision 10 December 2014

Thank you for submitting your revised manuscript for The EMBO Journal. Your study has now been seen by two of the original referees (comments included below) and as you will see they both find that all main criticisms have been adequately addressed and therefore support publication. However, before we can proceed to officially accept your manuscript and transfer the files for production I have to ask you to address the following editorial points in a final revision of the manuscript.

-> Please provide short paragraphs stating author contributions and conflict of interest. Please also reset the colour scheme for the word file to remove the yellow highlight of text added in revision.

-> Please fill out and include the author checklist as described in our online guide to authors. The checklist can be downloaded from http://emboj.embopress.org/authorguide.

-> We encourage the publication of source data, particularly for electrophoretic gels and blots, with the aim of making primary data more accessible and transparent to the reader. Would you be willing to provide files comprising the original, uncropped and unprocessed scans of all gels used in the figures? We would need 1 file per figure (which can be a composite of source data from several panels) in jpg, gif or PDF format. The gels should be labelled with the appropriate figure/panel number, and should have molecular weight markers; further annotation would clearly be useful but is not essential. These files will be published online with the article as a supplementary "Source Data".

-> As of Jan 1st 2014 every paper published in The EMBO Journal includes a 'Synopsis' to further enhance its discoverability. The synopsis consists of a short standfirst - written by the handling editor - as well as 2-5 one sentence bullet points that summarise the paper and are provided by the authors. I would therefore ask you to include your suggestions for bullet points.

-> I would also encourage you to provide an image for the synopsis. This image should provide a rapid overview of the question addressed in the study but still needs to be kept fairly modest since the image size cannot exceed 550x400 pixels.

Thank you again for giving us the chance to consider your manuscript for The EMBO Journal, I look forward to your final revision.

REFEREE REPORTS

Referee #1:
The authors have addressed all my concerns from the previous review.

Referee #3:
The authors have addressed the queries and replied satisfactorily, as far as this referee is concerned may be published as it is.

© European Molecular Biology Organization
