# Supplementary figures and images for: Nuclear matrix protein Matrin3 regulates alternative splicing and forms overlapping regulatory networks with PTB
Source: EMBO J. 2015 Jan 19;34(5):653–68. doi: 10.15252/embj.201489852 (PMC4365034; doi:10.15252/embj.201489852)

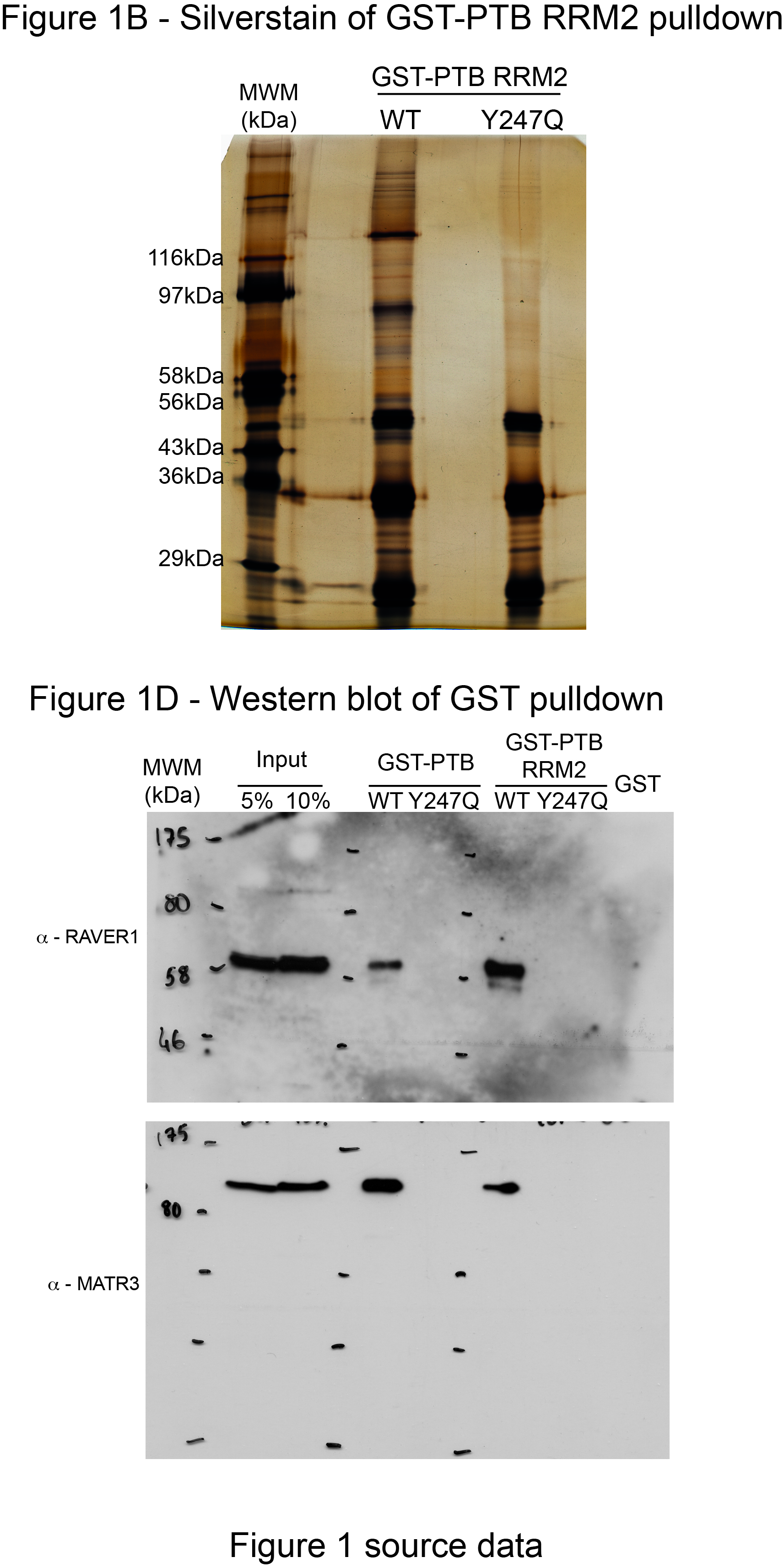

Supplement: Supplementary file 8 [file embj0034-0653-sd8.tif]

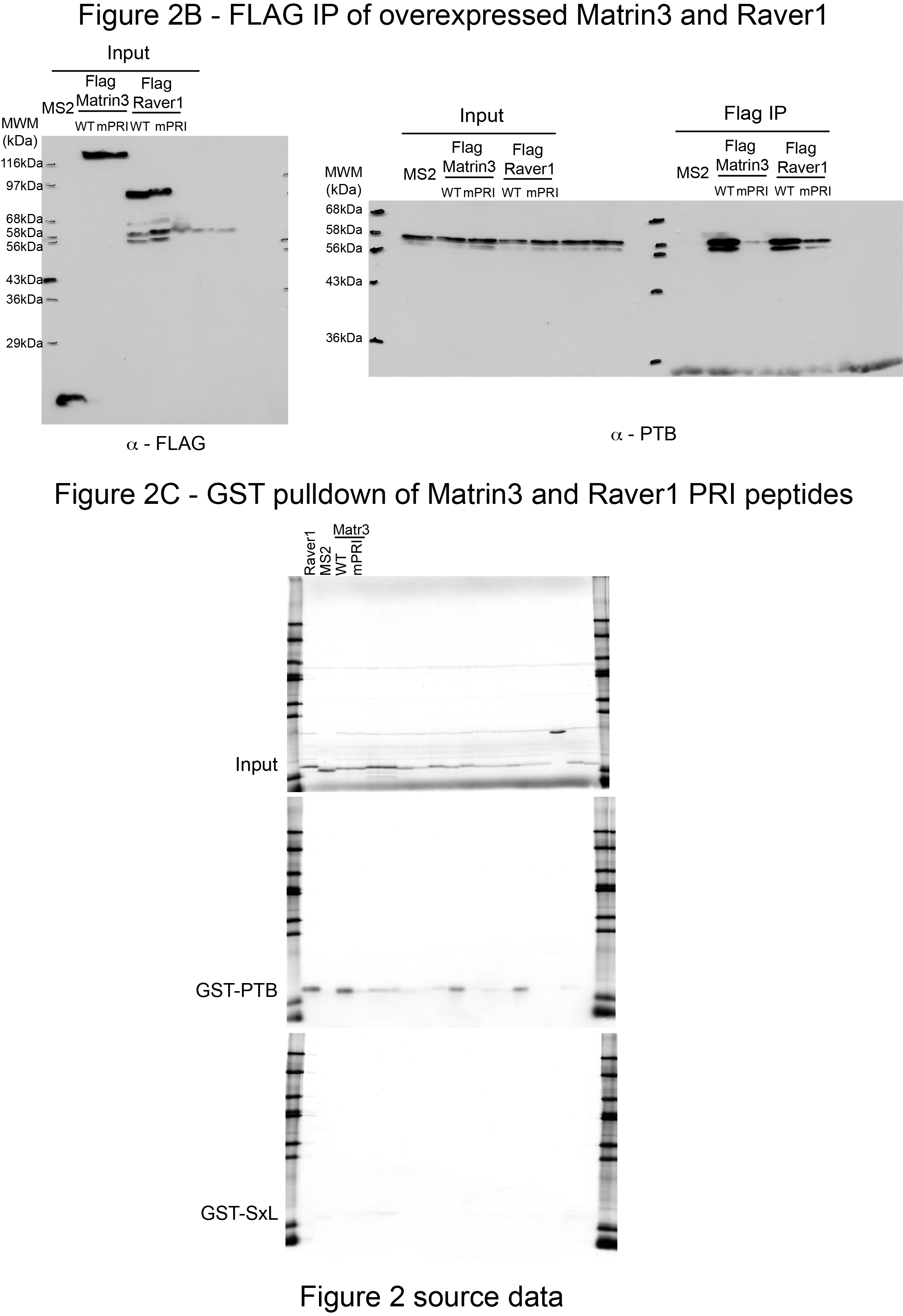

Supplement: Supplementary file 9 [file embj0034-0653-sd9.tif]

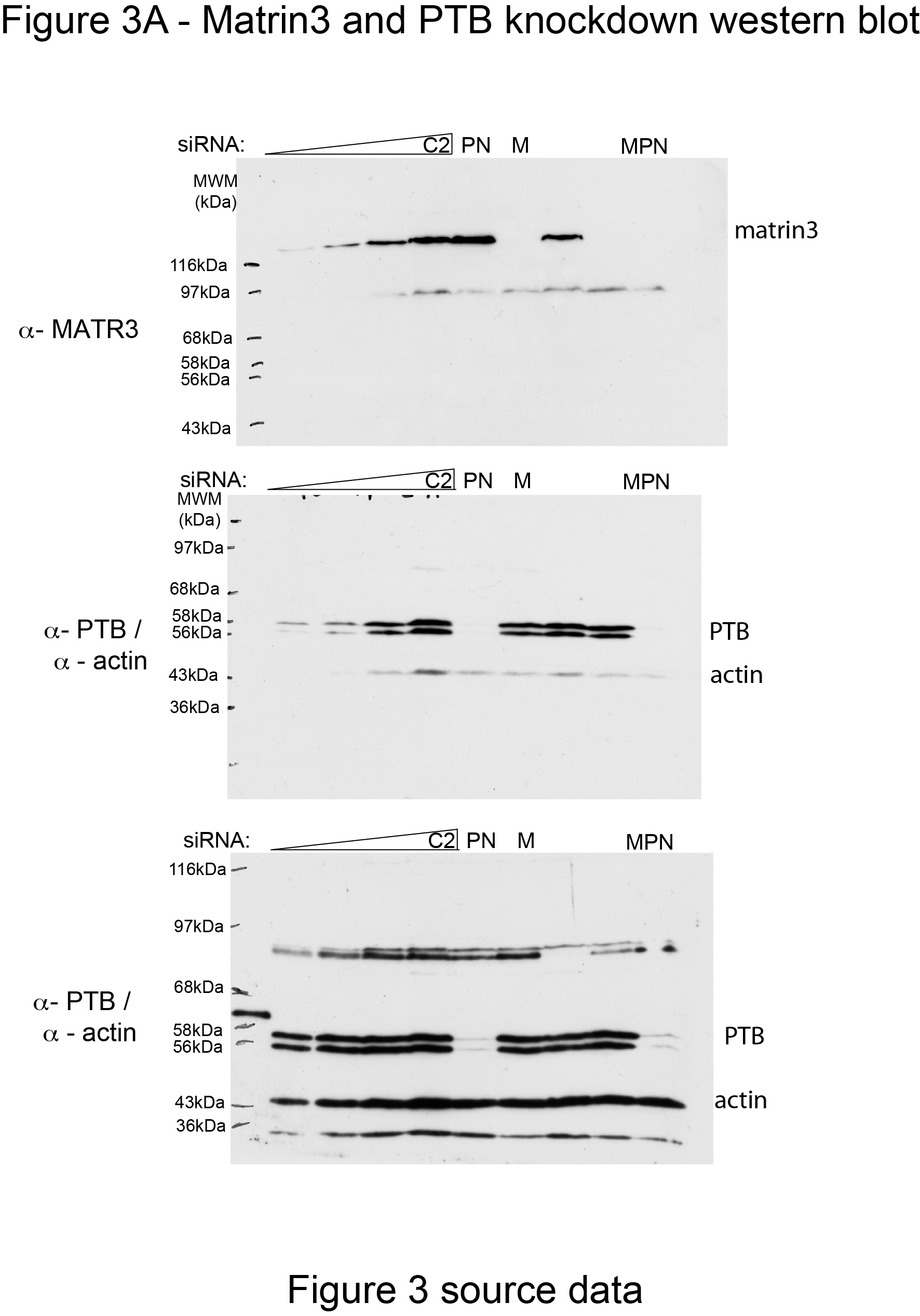

Supplement: Supplementary file 10 [file embj0034-0653-sd10.tif]

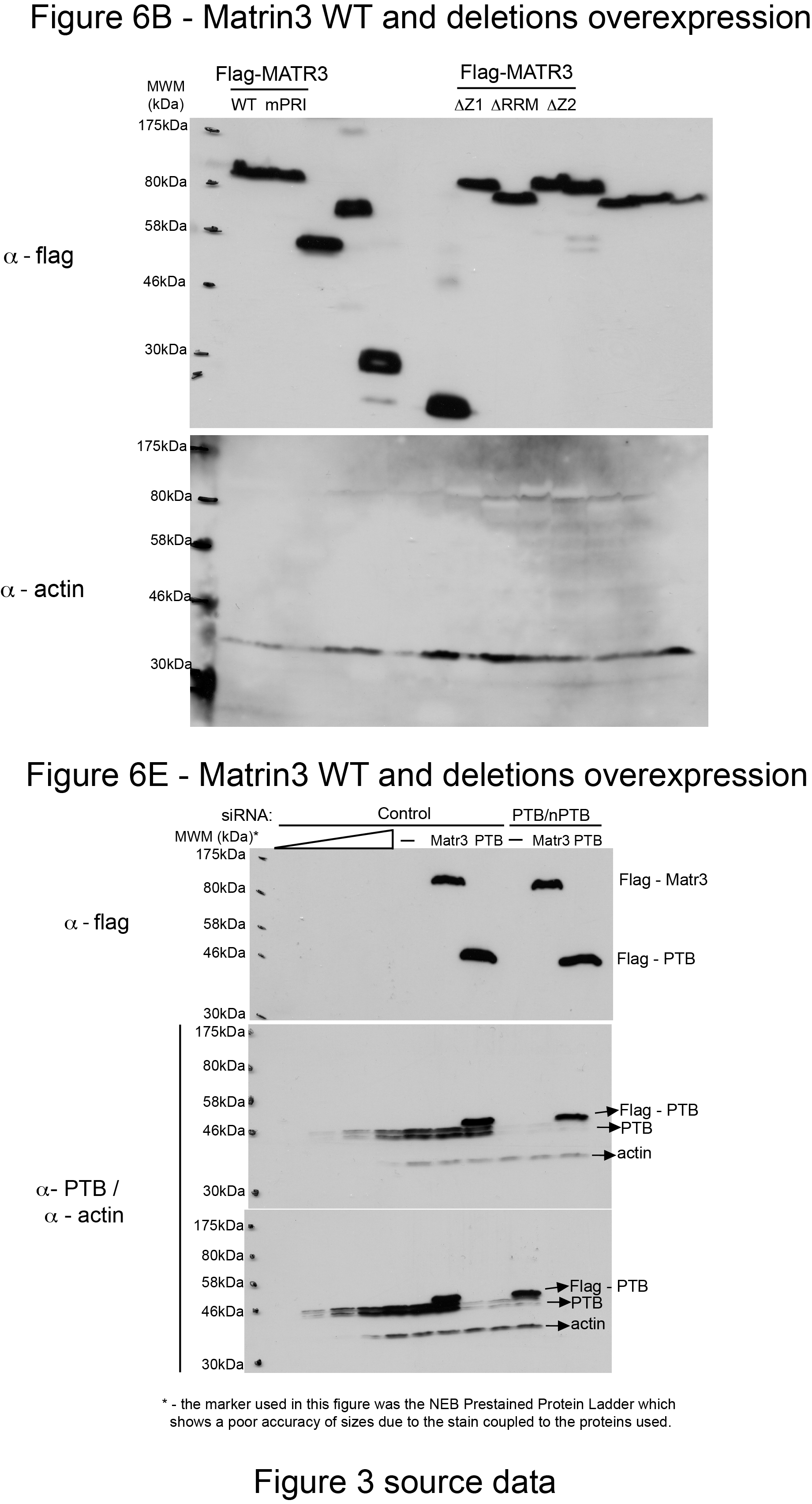

Supplement: Supplementary file 11 [file embj0034-0653-sd11.tif]
